# Variable number tandem repeats mediate the expression of proximal genes

**DOI:** 10.1038/s41467-021-22206-z

**Published:** 2021-04-06

**Authors:** Mehrdad Bakhtiari, Jonghun Park, Yuan-Chun Ding, Sharona Shleizer-Burko, Susan L. Neuhausen, Bjarni V. Halldórsson, Kári Stefánsson, Melissa Gymrek, Vineet Bafna

**Affiliations:** 1 Department of Computer Science & Engineering, University of California, San Diego, La Jolla, CA USA; 2 Department of Population Sciences, Beckman Research Institute of City of Hope, Duarte, CA USA; 3 Department of Medicine, University of California, San Diego, La Jolla, CA USA; 4 deCODE Genetics, Reykjavik, Iceland

**Keywords:** Computational biology and bioinformatics, Machine learning, Gene expression, Next-generation sequencing

## Abstract

Variable number tandem repeats (VNTRs) account for significant genetic variation in many organisms. In humans, VNTRs have been implicated in both Mendelian and complex disorders, but are largely ignored by genomic pipelines due to the complexity of genotyping and the computational expense. We describe adVNTR-NN, a method that uses shallow neural networks to genotype a VNTR in 18 seconds on 55X whole genome data, while maintaining high accuracy. We use adVNTR-NN to genotype 10,264 VNTRs in 652 GTEx individuals. Associating VNTR length with gene expression in 46 tissues, we identify 163 “eVNTRs”. Of the 22 eVNTRs in blood where independent data is available, 21 (95%) are replicated in terms of significance and direction of association. 49% of the eVNTR loci show a strong and likely causal impact on the expression of genes and 80% have maximum effect size at least 0.3. The impacted genes are involved in diseases including Alzheimer’s, obesity and familial cancers, highlighting the importance of VNTRs for understanding the genetic basis of complex diseases.

## Abstract (web summary)

Variable number tandem repeats (VNTRs) are implicated in human diseases yet have been difficult to analyse computationally. Here, the authors describe a neural network method, adVNTR-NN, that allows rapid and accurate genotyping of VNTRs from large whole genome sequencing datasets.

## Introduction

The human genome consists of millions of tandem repeats (TRs) of short nucleotide sequences. These are often termed as short tandem repeats (STRs) if the repeating unit is <6 bp, and variable number tandem repeats (VNTRs) otherwise. Together, they represent one of the largest sources of polymorphisms in humans^1,2^. While multiple resources have been developed for genome-wide analysis of STRs, here we focus specifically on VNTRs, which have been largely missing from genome-wide studies due to technical challenges of genotyping and the computational expense.

We define VNTR genotyping in the narrower sense of determining VNTR length (number of repeating units). As VNTRs can be located in coding regions^3^, untranslated regions (UTRs)^4^, and regulatory regions proximal to a gene^5,6^, the variation in length can have a significant functional impact. Not surprisingly, VNTRs have been implicated in a large number of Mendelian diseases that affect millions of people world-wide^7–9^. They also are known to modulate quantitative phenotypes in several other organisms^10^, and have shown pathogenic effects in other vertebrates including dogs^11^. VNTRs are also an important source of variations in bacteria and have commonly been used to study epidemiology and genetic diversity of *Mycobacterium tuberculosis* and *Yersinia pestis*^12,13^. They have influenced primate and human evolution through gene regulation and differentiation of great ape populations^14^. Recent studies have identified VNTRs that have expanded in the human lineage or are differentially spliced or expressed between human and chimpanzee brains^15^.

Single nucleotide polymorphisms (SNPs) that associate with gene expression, often referred to as expression quantitative trait loci (eQTLs), are molecular intermediates that drive disease and variation in complex traits^16–18^. Studies have shown that causal variants for diseases often overlap with *cis*-eQTL variants in the affected tissue^19^. Therefore, we focus on the specific application of identifying expression-mediating VNTRs (“eVNTRs”), or VNTRs located in regulatory regions whose length is correlated with the expression of a proximal gene. Examples of “eVNTRs” include a VNTR in the 5′ UTR of *AS3MT*, which is strongly associated with *AS3MT* gene expression and lies in a schizophrenia associated locus^4^ and a 12-mer expansion upstream of the cystatin B (*CSTB*) gene is associated with gene expression and with progressive myoclonus epilepsy^9,20^.

Despite their importance, the full extent of VNTRs in mediating Mendelian and complex phenotypes is not known due to genotyping challenges. Traditionally, VNTR genotyping used capillary electrophoresis which did not scale to large cohorts. Despite the advent of sequence based genotyping, repetitive sequences continue to be challenging for genomic analysis. For example, “stutter errors” due to polymerase slippage during PCR amplification change VNTR length and reduce genotyping accuracy^1^. While tools for genotyping STRs have been developed^1,21,22^, they generally do not detect or genotype VNTRs, which have non-identical and larger repeat units. Recently, a few specialized computational methods (including our own method, adVNTR) have been published to tackle the problem of genotyping VNTRs from sequence data^23,24^. However, these methods are too computationally intensive to scale to functional studies with hundreds of individuals and 10^4^ VNTR loci (Results). There have also been recent, successful efforts to genotype VNTRs using long-read sequencing technologies such as Pacific Biosciences (PacBio) and Oxford Nanopore Technologies (ONT)^23,25,26^. While these methods (which include adVNTR) are quite accurate, the technologies are currently too expensive for population scale sequencing.

For these reasons, large-scale studies of VNTRs and their association with gene expression have been limited when compared to other sources of human variation such as SNPs and CNVs^19,27,28^. While the standard whole genome sequencing (WGS) frameworks often ignore repetitive regions, there is some progress towards “harder” variant classes such as eSTRs^29–31^ and “eSVs”^28^. Therefore, “missing heritability”—the gap between estimates of heritability, measured for example by twin studies^32,33^, and phenotypic variation explained by genomic variation—remains a limitation for eQTL studies^34^. It has been speculated that the inclusion of TRs in association analyses may reduce this heritability gap^7,34,35^.

Here, we describe adVNTR-NN, a method that uses shallow neural networks for fast read recruitment followed by sensitive Hidden Markov Models (HMMs) for genotyping. We test the speed and accuracy of adVNTR-NN on extensive simulations to demonstrate accuracy. We use adVNTR-NN to genotype over 10,000 VNTRs in 652 individuals from the GTEx project and associate VNTR length with gene expression in 46 tissues. We additionally validate eVNTRs in blood tissues in 903 samples from an Icelandic cohort and 462 samples from the 1000 genome project with gene expression data (Geuvadis cohort). We compare the strength of genic eVNTR association against proximal SNPs and identified many of the eVNTRs as causal. Our results suggest that it is computationally feasible to genotype VNTRs accurately in thousands of individuals, and multiple eVNTRs are likely to causally impact the expression of key genes involved in common and complex diseases.

## Results

### Target VNTR Loci

**Fig. 1:**
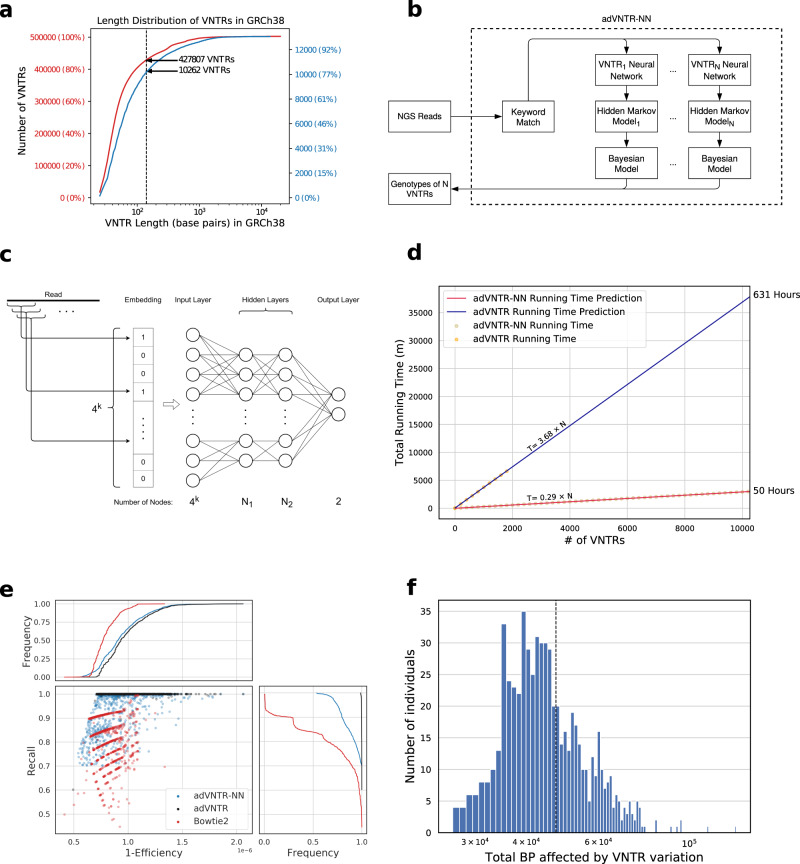
VNTR performance. **a** Length distribution of all known VNTRs (red) and selected targeted VNTRs (blue) across the GRCh38 human genome in base pairs. **b** The genotyping pipeline. **c** Neural network architecture for each VNTR which uses a mapping of reads to a k-mer composition vector. **d** Improvement in running time after using neural network and k-mer matching. **e** Accuracy and efficiency of read recruitment in simulated data. The scatter plot shows 1-efficiency ((TP + FP)/*R*) and recall (TP/(TP + FN)) of classification with different methods. High efficiency is related directly with running time. Each of 10,264 points represents a VNTR locus (Method) and are shown once for each method. The side and top panels show cumulative distributions of recall and 1-efficiency. **f** Base pairs (log-scale) affected by VNTRs per individual in the GTEx cohort. Source data are provided as a Source Data file.

Using Tandem Repeat Finder^36^, 502,491 VNTRs were identified that contained at least two repeating units in the GRCh38 human assembly and had repeat unit lengths between 6 and 100 bp. Over 80% of these had total length <140 bp (Fig. 1a) and could be genotyped using Illumina sequencing. As genotyping VNTRs remains computationally expensive, we focused on the 13,081 VNTRs located within coding, untranslated, or promoter regions of genes (Methods) as they are most likely to be involved in gene regulation. Of those, we identified 10,262 VNTRs that were within the size range for short-read genotyping (Fig. 1a). We added two additional VNTRs that were previously linked to a human disease (Supplementary Data 1) to obtain 10,264 target loci^37,38^.

### adVNTR-NN improves genotyping speed

Our previously published tool, adVNTR, used customized HMMs for each VNTR and showed excellent genotyping accuracy, based on trio-analysis, simulations, and PCR^23^. However, HMMs are compute-intensive, and despite some filtering strategies used by adVNTR (Methods), the time to genotype *n* = 10 K VNTRs was about 631 h per individual. In developing adVNTR-NN, we first made significant improvements to pre-processing time. Next, we deployed a second filtering step with a two-layer feedforward network trained separately for each VNTR that accepted the k-mer composition for each read and filtered it specifically for that VNTR (Fig. 1b, c and Methods). The neural-network filter required 0.03 s per read, and filtered reads with high efficiency in filtering reads. For 55X WGS with *r* = 4.2 × 10^6^ unmapped reads, the NN supplied an average of 14 previously unmapped reads to each VNTR HMM. Combining with the mapped reads, each HMM received an average of 32 reads per VNTR locus. This reduced the running time for *n* VNTR loci to

1 $${T}_{\text{adVNTR-NN}}(n)=25.48+0.29n\,\text{mins. (Fig. 1d),}\,$$

allowing each individual to be genotyped at *n* = 10 K VNTRs in 50 CPU hours, a 13× speedup over adVNTR.

### adVNTR-NN outperforms alternative alignment methods at VNTRs

While adVNTR was highly accurate by itself, its final accuracy depended upon reads filtered for genotyping, and specifically on false negatives—reads that were incorrectly removed by a filter. Formally, a read sampled from a VNTR was considered to be true positive (TP) if it passed the filter for that VNTR, and false negative (FN) otherwise. False positives (FP)—reads that passed the filter despite not being from the VNTR locus—were a lesser concern because they would eventually be discarded by the HMM for not aligning well to the model. However, high false-positives increase the running time. To account for this, we measured the trade-off between efficiency (1 − (TP + FP)/*r*) and recall TP/(TP + FN).

For comparisons with alternative filters, we used Bowtie2 as a representative read-mapping tool^39^. These tools are designed for fast mapping of reads and are accurate for most of the genome, but are not specifically designed for VNTR mapping genotyping (could have high FN). As a second comparison, we used adVNTR^23^, which has high recall (low FN) for VNTR mapping. We used a mix of real and simulated reads to test performance (Methods).

In terms of efficiency (1 − (TP + FP)/*r*), Bowtie2 was the most efficient retaining only 0.9 in 10^6^ reads for further processing for 90% of the VNTRs. Both adVNTR and adVNTR-NN were slightly less efficient retaining about 1.2 reads per million for 90% of the VNTRs. However, they had significantly better recall. adVNTR-NN filtered reads with at least 90% recall for 99% of the target VNTR loci (Fig. 1e). In comparison, 80% of the loci achieved that recall for adVNTR, and only 27% of the loci had a recall of 90% for Bowtie2. Notably, adVNTR-NN had much better recall compared to adVNTR while also being more efficient, and therefore faster.

### adVNTR-NN speed and accuracy on simulated VNTR alleles

We had previously measured adVNTR genotyping accuracy^23^ using trio-consistency, comparison to long reads, and other methods. Similarly, we used a mix of WGS data and simulated reads (Methods) to measure adVNTR-NN accuracy.

The accuracy of VNTR genotyping using short reads depends critically on total allele length and length of repeat unit itself. adVNTR was 90% accurate on reads up to 90 bp in length, but its accuracy dropped subsequently (Supplementary Fig. S1). Similarly, its accuracy remained high for repeat unit length up to 40 bp, as long as the total allele length did not exceed the read-length (Supplementary Fig. S2). We reiterate that a majority of the known VNTRs have small allele length (Fig. 1a), and therefore the overall accuracy remains high.

Next, we compared the overall running time and accuracy of adVNTR-NN genotyping with VNTRseek^24^, which was not available at the time of original release of adVNTR. Notably, VNTRseek combines VNTR discovery and genotyping and does not customize genotyping for each VNTR. Therefore, its running time on 55X WGS ranged from 9640–9686 min, and was largely independent of the number of target VNTRs (Supplementary Fig. S3). This was in contrast to the 1696 min required by adVNTR-NN. The speed advantage for adVNTR-NN could largely be attributed to filtering strategies which could potentially be used to improve VNTRseek genotyping time as well. On simulated heterozygous reads with 30X coverage (Methods), adVNTR-NN was highly accurate. It achieved 100% accuracy in 7343 (76%) of 9638 VNTRs compared to VNTRseek’s median accuracy of 60% (Supplementary Fig. S4). In contrast with adVNTR-NN, VNTRseek’s genotyping accuracy was sharply asymmetric, with much lower accuracy for decreasing VNTR length (Supplementary Fig. S5).

GangSTR is a method designed for STRs, but can genotype repeat units up to 20 bp^40^. Therefore, we compared its accuracy against adVNTR-NN for these short motifs. GangSTR uses total allele length which could result in an incorrect call if there are significant changes in repeat unit length. Indeed, on reference data, GangSTR was accurate in 82.4% of the VNTR loci and under-counted the allele by 1 in 12.3% of the cases. adVNTR-NN called the genotype correctly in 98.5% of the loci (Supplementary Data 2, 3). On simulated heterozygous reads, GangSTR accuracy lagged that of adVNTR-NN (Supplementary Fig. S6).

### adVNTR-NN consistency on trio data

A reference database of VNTR allele counts is not available for testing performance on real data. Instead, we tested for consistency of adVNTR-NN calls on 10,264 VNTRs using WGS data of 537 trios from 1000 Genomes Project^41^ (5,511,768 tests total). We observed 98.4% consistency in the calls obtained by adVNTR-NN. The inconsistent alleles had longer length (median 90 bp) in contrast to the length of the consistent alleles (median 52 bp, Supplementary Fig. S7) a range in which VNTR genotyping is more likely to be erroneous. Moreover, in a third of the inconsistent cases (0.5% of total), the RU count of the inconsistent allele was ±1 of a parent’s RU count, suggestive of a de novo mutation. Comparing adVNTR-NN genotypes with adVNTR, the calls were identical in 99.81% of the loci showing high similarity in accuracy between two genotyping methods (Supplementary Data 4).

### Datasets for identifying eVNTRs

To identify expression-mediating VNTR loci (eVNTRs), we primarily used data from the GTEx project^19^ (Methods). The GTEx project provided WGS for 652 individuals as well as RNA-seq for each of these individuals from 46 tissue types including whole-blood. A majority (86.0%) of the donors were of European origin; another 11.5% were African American and the remaining were Asian and American Indian. For validation, we used a second cohort of 903 Icelandic individuals^42^ with associated whole blood RNA expression data and WGS. We also chose a smaller, third cohort from the Geuvadis^27^ project which provided gene-expression data in lymphoblastoid cell-lines for 462 samples, where the WGS for the samples was available from the 1000 genomes project^41^. The Geuvadis cohort was dominated by individuals of European ancestry (80.7% of cohort). Most of the remaining (19.3%) were of African ancestry. Due to the match of tissue type and ethnicity, the Icelandic and Geuvadis whole blood data were used for validation of methods for identifying eVNTRs discovered from the GTEx project.

### eVNTR identification

We genotyped 10,264 VNTR loci in all 652 samples from GTEx to study the role of VNTRs in mediating gene expression of proximal genes. As expected, the most frequent allele matched the reference allele in 96.8% of the cases (Supplementary Fig. S8).

**Fig. 2:**
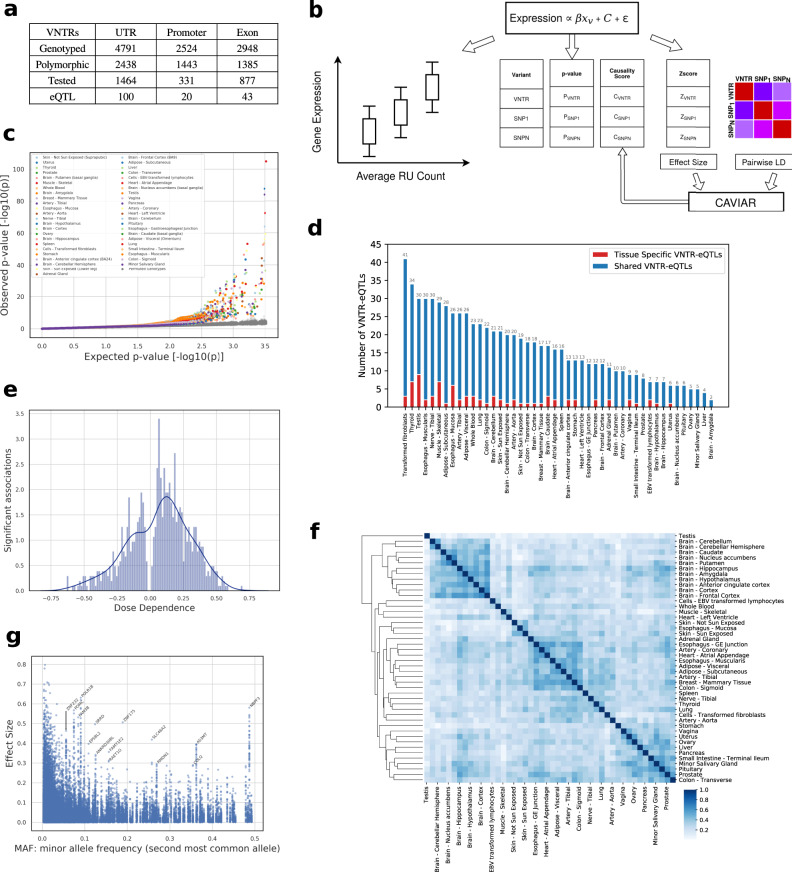
Effect of VNTR genotypes on mediating gene expression. **a** Location of target VNTRs and eVNTRs relative to the proximal genes. **b** Pipeline to identify eVNTRs and assign causality scores. Ancestry, Sex, and PEER factors are included in *C* as covariates. We associate VNTR genotype with expression residuals after correcting for the effect of *C*. **c** Quantile-quantile plot showing *p* values of association signals separated by tissue. Green line represents the *p* values using 100 permutations. **d** Number of unique and shared eVNTRs in each tissue. **e** Trend of RU count correlation with gene expression level. **f** Spearman correlation of eVNTRs effect sizes for each pair of tissues. **g** Scatter plot correlating effect size versus minor allele frequency (MAF). Source data are provided as a Source Data file.

Despite the GTEx data being predominatly European, 51% of the target VNTRs were polymorphic. Consistent with evolutionary constraints, VNTRs in promoters were most likely to be polymorphic (57%) followed by UTRs (51%) and coding exons (47%) (Fig. 2a). Each individual in the GTEx cohort had a non-reference allele in at least 839 (8.2%) of the tested VNTR loci, with an average of 1259 (12.3%) non-reference VNTRs per individual. Altogether, the 10,264 VNTRs inserted or deleted an average of 47,197 bp per individual (Fig. 1f). As this represents <10% of all VNTRs, the results highlight VNTRs as an important source of genomic variation. The minimum variation in a non-reference VNTR allele involved at least 6 bp and the average change in each variant site was 37 bp or about 3 repeat units (Supplementary Fig. S9).

To perform association analysis, we excluded 1817 (17.7% of total) VNTRs that were monomorphic, 1445 (14.1%) VNTRs that violated Hardy–Weinberg equilibrium constraints, and 4330 (42.2%) VNTRs that had minor allele frequency (MAF) <1% after removing individuals in the GTEx cohort with no expression data for the specific gene (Methods). We investigated VNTRs that violated HWE. Similar to trio-inconsistent VNTRs but distinct from all VNTRs, these VNTRs were longer, had long common alleles (Supplementary Figs. S10 and S11), or their flanking regions had a strong (>5 bp) match to the sequence of the repeating units (Supplementary Fig. S12).

The filtering resulted in a set of 2672 VNTRs (26%) available for association analysis. We used linear regression to measure the strength of association between average VNTR length of the two haplotypes, and adjusted gene expression level of the closest gene (Fig. 2b and Methods). To account for confounding factors, we included sex and population principal components (PCs) of each individual as covariates. We also added PEER (probabilistic estimation of expression residuals) factors to account for experimental variations in measuring RNA expression levels (e.g., batch effects, environmental variables)^43^. Briefly, PEER infers hidden covariates influencing gene expression levels, and we removed their effect by producing a residual gene expression matrix and using it for linear regression (See Methods).

We measured association with gene expression in each of the 46 tissues. To control false discovery rate (FDR), we used the Benjamini–Hochberg procedure to identify a tissue-specific 5% FDR cutoff (Supplementary Fig. S13 and Methods). Combining data from all tissues, 759 tests tied to 163 unique VNTR loci passed the significance threshold (Fig. 2c). We refer to these (VNTR, gene) pairs as eVNTRs. Unlike VNTRs that failed HWE (median length: 92 bp), eVNTR allele lengths were much smaller (median:48 bp, Supplementary Fig. S10), and in a range where VNTR genotyping is highly accurate (Supplementary Fig. S1).

Not surprisingly, a larger fraction (6.8%; Fig. 2a) of the UTR and regulatory (6.0%) variants were associated, compared to coding VNTRs (4.9%). The strength of association did not depend upon the location of the VNTRs (Supplementary Fig. S14). However, VNTRs within 100 bp of the transcription start sites (TSS) were twice as likely to be eVNTRs compared to other locations (*P* = 6 × 10^−6^; Fisher’s exact test), consistent with their known roles in core-promoters^44^.

The number of eVNTRs observed in each tissue type generally correlated with the number of individuals samples for each tissue type (Supplementary Fig. S15). Consistent with previous results on eQTLs^19^, and eSTRs^31^, testis and fibroblasts had the largest number of eVNTRs, while fewer eVNTRs were identified in whole blood and skeletal muscle, relative to the sample size. Only 4% of the eVNTRs were tissue-specific (Fig. 2d). We used the method mash^45^ to test for reproducibility in other tissues. Mash exploits the power gains that come from cross-sharing the effect of an eVNTR in multiple tissues. The analysis suggested that many (38%) eVNTRs were significant in at least half (23) of the tissues tested (Supplementary Fig. S16).

Twenty-three of the 163 unique eVNTRs showed significant association in whole blood (Table 1), a tissue type in which we could validate the eVNTRs using independent data from the Icelandic cohort of 903 individuals. The VNTRs that showed significant associations in GTEx were replicated on the Icelandic cohort using the conservative *p* value cutoff from the smaller GTEx cohort. Two of the 23 VNTR loci could not be used for replication in the Icelandic cohort due to missing expression data for *TRIM15* and *SNHG16* genes. Of the 21 VNTRs, 18 (86%) showed significance at a similar level and same direction of effect in Icelanders, highlighting the strong reproducibility of the associations. The Geuvadis data were acquired for a smaller cohort compared to the Icelandic data and measured expression in lymphoblastoid cells–transformed B cells, which are a component of whole blood tissue. Therefore, we recomputed 5% FDR cut-offs using the Benjamini–Hochberg method on 100 permuted samples. Despite the caveats, 12 of the eVNTRs were replicated. Combined, 91% (20/22) of eVNTRs could be replicated in an independent cohort where data was available. We also tested for correlation of effect sizes between the Icelandic and GTEx data and found strong correlation (Supplementary Fig. S17; Spearman correlation coefficient 0.88; *p* value = 1.15 × 10^−7^). A similarly strong correlation was observed between the Geuvadis cohort and GTEx (Supplementary Fig. S18; Spearman correlation coefficient 0.70; *p* value: 4.57 × 10^−4^). In all cases, the direction of effect was also maintained.

**Table 1:** Replication of whole blood VNTRs in independent cohorts.

							Replication	
	Locus	Length	RU Length	Effect Size	Gene	Annotation	Icelandic	Geuvadis
1	chr1:21440112–21440147	35	6	0.43	*NBPF3*	UTR	Y	Y
2	chr2:24084339–24084414	75	25	−0.12	*TP53I3*	UTR	Y	Y
3	chr2:25161573–25161616	43	9	0.22	*POMC*	Coding	Y	Y
4	chr2:112542424–112542500	76	25	−0.18	*POLR1B*	Coding	Y	Y
5	chr3:56557249–56557289	40	20	−0.12	*CCDC66*	Coding	Y	Y
6	chr6:13328502–13328532	30	6	0.12	*TBC1D7*	UTR	Y	Y
7	chr7:64337190–64337240	50	13	0.09	*ZNF736*	UTR	Y	Y
8	chr8:86508719–86508765	46	23	0.13	*RMDN1*	UTR	Y	Y
9	chr10:102869497–102869605	108	36	0.22	*AS3MT*	Coding	Y	Y
10	chr21:46228815–46228863	48	9	−0.03	*LSS*	UTR	Y	Y
11	chr17:75589192–75589228	36	6	−0.06	*MYO15B*	Coding	Y	-
12	chr1:46609102–46609134	32	16	0.09	*MOB3C*	UTR	Y	N
13	chr5:80654880–80654954	74	9	0.04	*MSH3*	Coding	Y	N
14	chr9:137063433–137063550	117	39	−0.15	*SAPCD2*	UTR	Y	N
15	chr14:61762420–61762454	34	17	0.03	*SNAPC1*	UTR	Y	N
16	chr19:12577507–12577551	44	22	−0.09	*ZNF490*	UTR	Y	N
17	chr21:41316673–41316756	83	13	−0.19	*FAM3B*	UTR	Y	N
18	chr22:37805258–37805313	55	6	0.11	*H1F0*	UTR	Y	N
19	chr1:202187007–202187042	35	7	0.06	*PTPRVP*	UTR	N	Y
20	chr17:18208488–18208544	56	7	−0.13	*ALKBH5*	UTR	N	Y
21	chr17:76564106–76564152	46	9	0.11	*SNHG16*	UTR	-	N
22	chr17:56978047–56978107	60	20	0.15	*SCPEP1*	UTR	N	N
23	chr6:30163542–30163579	37	12	0.14	*TRIM15*	UTR	-	-

Each row describes an eVNTR in whole blood from GTEx project(*n* = 652 individuals) identified with false discovery rate (FDR) <0.05 based on 100 permutations. Replication of the signal in whole blood tissue of the Icelandic cohort of 903 samples and in lymphoblastoid cell-lines from the Geuvadis cohort (462 samples) with the same direction of effect and FDR <0.05. For the Icelandic cohort, only the VNTRs that showed significant associations in GTEx were tested using unmapped reads plus reads mapped to those specific loci. Hence, we used the conservative *p* value cutoff from the smaller GTEx cohort. Length (respectively, RU length) refers to the total (respectively, repeat-unit length) of the VNTR.

STR genotyping software such as HipSTR^1^ can also genotype repeats up to 6 bp. Therefore, we compared GTEx association results on hexamer repeats from a recent eSTR study^31^. Fifteeen loci were identified as eSTR/eVNTR in at least one of the two studies (Supplementary Table S1). Despite differences in genotyping methods, filtering, FDR controls, choice of covariates, and reference assemblies, all 15 loci were at least nominally significant in both tests, and 6 of 15 were identified as eSTRs/eVNTRs in both studies.

In 65% of the cases, VNTR length had a positive correlation with gene expression; the remaining cases had a negative correlation (Fig. 2e). This was consistent with the hypothesis that many VNTRs encode transcription factor binding sites and increasing length improved the TF binding affinity. Moreover, the overall effect size was also large and 80% of the eVNTRs had a maximum effect size 0.3 or higher.

We computed correlation of eVNTR effect size between each pair of tissues using the Spearman rank test. Despite the multi-tissue activity of most eVNTRs, each tissue showed distinct behavior with low correlation to most other tissues (Fig. 2f). Similar tissue types were expectedly correlated (e.g., brain). Some correlations were seen among glandular tissues (salivary, prostate, and pituitary) and also between adipose tissue and nearby tissues and organs (heart, esophagus muscularis, artery, and breast). Fotsing et al.^31^ used eSTRs to cluster a subset of 17 tissue types. When restricted to that subset (Supplementary Fig. S19), the eVNTR clustering was highly consistent with the eSTR clustering. Both analyses showed distinct clades for (a) the two skin tissues with esophageal-mucosa possibly due to an abundance of squamous cells and (b) the two adipose tissues with esophageal-muscularis. Moreover the second clade was part of a larger one containing the arterial tissues, the tibial nerve, thyroid, and lung in both analyses. Thus, even though most eVNTRs are shared across tissues, we hypothesize that the combined effect of active eVNTRs is tissue-specific and leads to unique regulatory program for each tissue type.

Similar to SNPs, and due in part to power considerations, VNTR loci generally showed a negative correlation between MAF and effect size, so that common variants generally had low effect size with larger effects mainly shown by rare variants^46^ (Fig. 2g). However, we still observed many eVNTRs where common VNTR (MAF >0.05) showed large effects. These eVNTRs had highly significant *p* values (Supplementary Fig. S20) and in many cases, the proximal genes were associated with known diseases or phenotypes (Supplementary Data 5). As these represent potentially the most interesting eVNTR findings, we tested them further for causality and function.

### VNTRs mediate expression of key genes

Only a small number of examples have been reported where VNTR repeat unit counts have a causative on gene expression^4^. Each of these cases has been discovered by gel analysis or Sanger sequencing on individual loci in specifically chosen cohort. One well known example is the *AS3MT* gene which is involved in early brain development, where the VNTR was associated with expression and was in linkage disequilibrium (LD) with SNPs associating with schizophrenia^4^.

To investigate causality, we ranked each eVNTR against all SNPs within 100 kbp by (a) comparing the relative significance of association with gene expression (*r*_1_); and (b) using the tool CAVIAR^47^ to measure the causality of association (*r*_2_) (Methods). Remarkably, the two rankings were very similar with mean discrepancy 2∣*r*_1_ − *r*_2_∣/(*r*_1_ + *r*_2_) = 2.3 × 10^−3^ across the 163 eVNTRs. We used the harmonic mean $\left(2/\left(1/{r}_{1}+1/{r}_{2}\right)\right)$ of the two ranks to order the eVNTRs. Of the 163 VNTRs, 81 of the eVNTRs were ranked 1 which are likely causal (Supplementaary Fig. S21), indicating that the 49.6% of the eVNTRs had the highest posterior probability of causality compared to all other variants tested. Separating tissue types, 170 (22%) of the 759 significant associations were possibly causal. These results suggest a large fraction of causal eVNTRs even with the caveat that we only tested “genic” VNTRs.

**Fig. 3:**
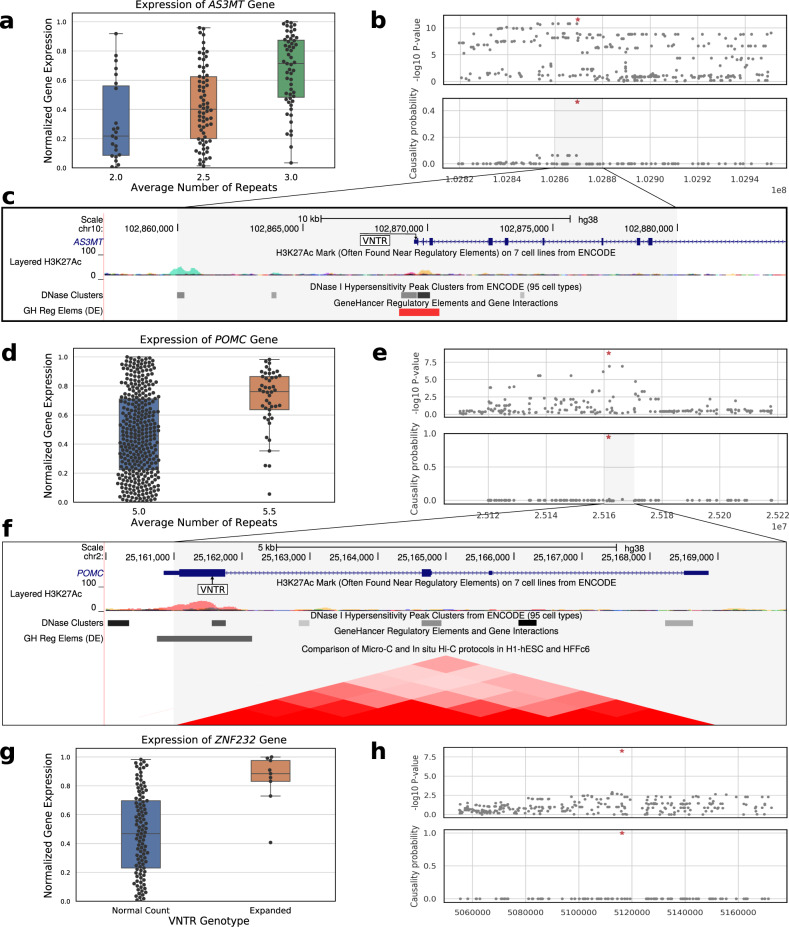
Effect of VNTR genotypes on mediating gene expression. **a** Association of *AS3MT* VNTR genotype with gene expression in brain cortex (*n* = 148 samples, Fisher’s two-sided *P*: 2.78 × 10^−12^). Box plots display the median, 25th and 75th percentiles. **b** Association with gene expression (upper panel) and CAVIAR causality probability of proximal SNPs—all SNPs in 100 kbp window on either side of the *AS3MT* VNTR (red-star). **c** Location of *AS3MT* VNTR relative to known regulatory elements. **d**, **e** Association with gene expression of the *POMC* VNTR (*n* = 378 samples, Fisher’s two-sided *P*: 1.53 × 10^−9^) and its causality probability relative to proximal SNPs. Box plots display the median, 25th and 75th percentiles. **f** Location of *POMC* VNTR relative to other regulatory regions and its spatial proximity with the promoter region revealed via Hi-C. **g**, **h** Association with gene expression of the *ZNF232* VNTR (*n* = 114 samples, Fisher’s two-sided *P*: 5.47 × 10^−9^) and its causality score relative to proximal SNPs. Box plots display the median, 25th and 75th percentiles. Source data are provided as a Source Data file.

Looking at individual eVNTRs, we recapitulated a previous result by identifying an eVNTR in the *AS3MT* gene. The lowest association *p* value measured in any tissue using 652 samples was 3.9 × 10^−54^, which was orders of magnitude higher than the significance reported with 322 samples^4^ (Fig. 3a, b). Its CAVIAR rank was 1 and it had an effect size of 0.33 in brain cortex in contrast to the effect size of 0.16 for the top SNP in brain cortex. Finally, the VNTR is located in a regulatory region of the genome as identified by H3K27Ac and DNase marks (Fig. 3c).

The other eVNTRs, including the 81 with CAVIAR rank 1, represent novel findings. Many mediate the expression of genes (Supplementary Data 5) involved in key functions. For example, proopiomelanocortin (*POMC*) is a precursor protein for many peptide hormones with multiple roles including regulation of appetite and satiety^48^. Hypermethylation of *POMC* (and reduced expression) in peripheral blood cells and melanocyte-stimulating hormone positive neurons was strongly associated with obesity and body mass index^49^. Surprisingly, *POMC* over-expression also predisposed lean rats into diet-induced obesity^50^. Our analysis identified a VNTR in the coding region of the *POMC* gene as the causal variant governing expression levels in 15 tissues, including adipose and nerve tissues. The 6R allele had 1.8-fold higher expression in blood and nerve cells (Fig. 3d), and the correlation with expression was much stronger than neighboring SNPs (Fig. 3e). The eVNTR had an effect size of 0.48 in Nerve tissue, compared to 0.27 for the top SNP using the same model. Moreover, the VNTR was located within an H3K27Ac mark that was topologically close to the promoter of the gene based on chromatin conformation (Fig. 3f).

The *ZNF232* gene is differentially expressed in ovarian and breast cancers^51,52^. Also, the chr17 locus containing the gene has been associated with Alzheimer’s in a recent large meta-GWAS study on the UK Biobank data^53^. We identified an eVNTR in the promoter region where expanded alleles (at least 5 repeat units) had 2-fold higher median expression relative to RU3 (Fig. 3g). The VNTR was ranked 1 in 40 of 46 tissues including seven brain sections, and specifically the hippocampus, which is the affected region in Alzheimer’s^54,55^ (Fig. 3h) and was also ranked 1 in ovary and breast tissues (Supplementary Data 5). In hippocampus, the eVNTR effect size was 0.34 for eVNTR compared to 0.07 for top SNP using the same model.

The *RPA2* gene product is part of the replication protein A complex involved in DNA damage checkpointing^56^. Its over-expression is identified as a prognostic marker for colon and bladder cancers^57^. A VNTR that overlapped the TSS of *RP2A* with lower VNTR length showed 1.9-fold higher expression of *RPA2* in multiple tissues including colon (Supplementary Fig. S22 and Supplementary Data 5). Supplementary Data 5 identifies other important genes including *NBPF3* (neuroblastoma^58^), *TBC1D7* (lung cancer^59^), *ZNF490* (colorectal cancer^60^), *MSH3* (myotonic dystrophy^61^). We note that the VNTR in *MSH3* is a 9 bp repeat that is distinct from the trinucleotide expansion mediated by *MSH3*^62^. Taken together, our results suggest that VNTRs mediate the expression of key genes.

## Discussion

VNTRs are the “hidden polymorphisms.” Despite high mutation rates and known examples of function modifications, VNTR genotyping is not a component of Mendelian or GWAS pipelines. This is primarily due to technical challenges. Here, we use a combination of fast filtering followed by a HMM-based genotyping to accurately determine VNTR genotypes. Our method, adVNTR-NN, can genotype 10K VNTRs for an individual in 50 cpu hours with high accuracy. We used adVNTR-NN to genotype close to 2000 human samples at 10K loci. The use of neural networks as a filtering strategy is novel, and we believe that further improvements could lead to another order of magnitude reduction in compute time, making it practical to genotype ≥10^5^ individuals in the future.

Some VNTRs have complex multi-repeat structure making it difficult to map reads and count the repeating units. However, unlike other VNTR genotyping methods, our method customizes the genotyping for each VNTR. Future research will focus on improving the genotyping for the hard cases, possibly by building HMMs with separate profiles for each distinct repeating unit, as well as the use of long-reads to improve anchoring to the correct locations. We pursue a targeted genotyping approach which has the disadvantage of not being able to discover new VNTRs, and we rely on other methods for the initial discovery of VNTRs. However, we note that the discovery is a one-time process while genotyping must be repeated for each cohort, and therefore, it makes sense to separate the two problems. For maximum sensitivity, discovery of VNTRs could be performed on a new cohort prior to genotyping. Even if the reference contained 0 copies, knowledge of the repeat pattern and location would allow us to genotype donors with multiple repeat units.

The relatively large number of VNTRs violating HWE suggests that genotyping accuracy could be improved by filtering problematic VNTRs. We are developing strategies to filter VNTRs based on similarity to other VNTRs, matching sequence of repeat units and flanking regions, and other tests for long alleles. As more data is collected, we will be able to assess the accuracy of these strategies.

adVNTR-NN can be used for association of a VNTR genotype chosen from a large collection of target VNTRs, against categorical or quantitative phenotypes. We used it to identify eVNTRs, where VNTR allele changes associated strongly with gene expression. It is possible that the largest allele or some other regrouping has the strongest effect for some VNTRs, and this idea may be used to strengthen the eVNTR association. However, we did not have a consistent strategy for grouping the VNTRs and therefore did not try this approach for the VNTRs in our study. Nevertheless, for individual VNTRs that are on the borderline for significance, this approach could be tried prior to functional tests.

We found that VNTRs were strongly associated with the expression of proximal genes with over 6.1% of the 2672 VNTRs tested showing genome wide significant association. Nearly half of the eVNTR loci were more significant compared to neighboring SNPs. While the high fraction of causal eVNTRs can partly be explained by the choice of “genic” VNTRs for testing, we believe that non-genic regions will identify additional causal eVNTRs. In testing for causality, it would be best to compare against all other forms of variation including SNPs (which include small indels), structural variations, and other STRs. However, there is significant complexity in calling these variants. For example many STRs and even VNTRs are mis-annotated as structural variants. We will address these concerns in future work. In summary, ongoing technical innovations in speed and accuracy of VNTR genotyping are likely to improve our understanding of human genetic variation, and provide novel insights into the function and regulation of key genes and complex phenotypes.

## Method

### Genotyping in adVNTR-NN

#### Filtering trade-off calculations

Let *A*(*r*) denote the HMM genotyping time using *r* reads. The goal of filtering is to reduce the number of reads supplied to each VNTR HMM. Any filter is characterized by three parameters:

run-time: Let *P*(*r*) denote the running time of the filter for *r* reads for each VNTR locus;

efficiency: Let *f*_*k*_ denote the fraction of reads that were retained for any VNTR. The efficiency is defined as 1 − *f*_*k*_ so that high efficiency implies only a small fraction being retained by the filter.

sensitivity/recall: The fraction of true VNTR overlapping reads that were accepted for each VNTR.

Consider a dataset with *r* unmapped reads and among the mapped reads, an average of $r^{\prime}$ reads are assigned to each VNTR locus. Assuming that the filtered reads are distributed equally among the VNTRs, each HMM will receive ${f}_{k}r+r^{\prime}$ reads on the average. The total genotyping time for *n* VNTRs is given by:

2 $${T}_{\text{adVNTR}}(n,r,r^{\prime} )=\,{\text{indexing-time}}\,+n\left(\right.P(r)+A\left({f}_{k}r+r^{\prime} \right)\left)\right.,$$

Empirically, *A*(*r*) = 0.32*r* seconds per VNTR. The keyword match filter for adVNTR achieved *f*_*k*_ = 7.7 × 10^−5^. For a 55X coverage WGS with *r* = 4.2 × 10^6^ reads, *P*(*r*) = 111.22(*s*), $r^{\prime} =18$, we run the HMM on an average of ${f}_{k}r+r^{\prime} =341$ reads per VNTR on the average. The running time is:

3 $${T}_{\text{adVNTR}}(n,r)=60.23+n\left(1.853+\frac{0.32}{60}\times 7.7\times 1{0}^{-5}\times 4.2\times 1{0}^{6}+\frac{0.32}{60}\times 18\right)$$

4 $$=60.23+3.68n\,\text{mins.}\,,$$

The genotyping time for *n* = 10K VNTRs is about 631 h per individual.

#### Read filtering

For each VNTR locus *V*, and each read *R*, consider a binary classification function *f*: *V* × *R* → {0, 1}, where *f*(*R*, *V*) = 1 if and only if read *R* maps to locus *V*. For each read and each of *N* loci *V*_1_, …, *V*_*N*_, the neural recruitment method computes independent classification functions *f*_i_(*V*_i_, *R*). Note that a read can be assigned to multiple VNTR loci, or to none. As an initial step toward this task, we perform a fast string matching based on prefix tree (trie) to assign each read to the VNTR loci that share an exact match with the read. For an efficient matching, we generate a separate aho-corasick trie^63^ using every k-mer in VNTR loci as dictionary *X*. A trie is a rooted tree where each edge is labeled with a symbol and the string concatenation of the edge symbols on the path from the root to a leaf gives a unique word (k-mer) *X*. We label each leaf with a set of *T* VNTRs that contain corresponding k-mer. On the other hand, the string concatenation of the edge symbols from the root to a middle node gives a unique substring of X, called the string represented by the node. We add extra internal edges called failure edges to other branches of the trie that share a common prefix which allow fast transitions between failed string matches without the need for backtracking^63^. Testing whether a query *q* has an exact match in the trie can be done in *O*(∣*q*∣) and we require additional *O*(∣*T*∣) time to assign read *q* to all *T* VNTR loci that share the keyword. The overall complexity of this algorithm is linear based in the length of original dictionary (VNTRs in the database) to build the trie and recover matches plus the length of queries (sequencing reads). Hence, after construction of the trie, the running time is proportional to just reading in the sequences.

#### Neural recruitment

To further reduce the set of reads assigned to each VNTR, we use a 2-layer feedforward neural network to compute *f*_i_, using a k-mer based embedding to encode DNA strings. Specifically, we use a DNA string *w* of length *k*, consider an bijection *ϕ* that maps *w* to a unique number in [0, 4^*k*^ − 1]. Each read *R* can be defined by a collection of overlapping k-mers. We map read *R* to a unique vector ${v}_{R}\in {\{0,1\}}^{{4}^{k}}$, such that *v*_*R*_[*i*] = 1 if and only if *ϕ*^−1^(*i*) ∈ *R*. Details of the neural network architecture and hyper-parameters are presented below.

#### Network architecture

Let *v* denote the mapping of a read. We use a shallow architecture with an input layer used to present *v* to the network. We add two layers of fully connected nodes as the hidden layers, with each node being a ReLU function. In the output layer, there are two nodes *zero* and *one* which specify that whether read should be classified as true (containing VNTR) or false (Fig. 1). We used the training set to train the network with Adam optimization algorithm^64^.

The number of hidden layers *N*_1_ and *N*_2_ were chosen empirically. Too many nodes would increase both training time and test time and possibly cause over-fitting. We performed the training with the number hidden nodes of each layer varying from 10 to 100 with 10 increase in each step and selected *N*_1_ = 100 and *N*_2_ = 50 as the best parameters according to validation performance.

#### Choosing the optimal k-mer length

The choice of k-mer length is important. Increasing the k-mer size could decrease sensitivity in our case as small variation will significantly change the k-mer composition, whereas lowering k-mer size reduces the features that are discriminative for a pattern^65^. In addition, our embedding size exponentially grows with respect to the *k* so there is also a practical upper bound on the *k*. Following Zhang^65^ and Dubinkina^66^, we trained and tested in the range 4 ≤ *k* < 9. The accuracy remains comparable in this range (Fig. S23), and we chose *k* = 6 as its mean validation accuracy is the highest compared to four other values of *k*.

#### Effect of different loss functions

To choose the best loss function, we examined three regression loss functions: Mean squared error (MSE), mean squared logarithmic error (MSLE), and mean absolute error (MAE), as well as three binary classification loss functions hinge, squared hinge, and binary cross-entropy. We compared the validation performance of our models for these six different loss functions. Each distribution in Supplementary Fig. S24 shows the accuracy on validation set across 1905 genomic loci. We analyzed these distributions using one-way analysis of variance (ANOVA) and none of them were significantly better than others. We chose binary cross-entropy as it obtained the highest mean accuracy (99.95%) among loss functions and its binary classification nature fits our requirement.

#### Speed and efficiency of neural network filtering

The neural-network filtering achieved a speed of *N*(*r*) ≃ 0.03*r* seconds for *r* reads, greatly increasing filtering efficiency (${f}_{n}{f}_{k}^{\prime}<1{0}^{-6}$) to input only 14 reads per VNTR on the average when *r* = 4.2 × 10^6^. The running time using the two filters could be modeled as

5 $${T}_{\text{adVNTR-NN}}(n,r) 	=n\left(P^{\prime} (r)+N\left({f}_{k}^{\prime}r\right)\right)+nA\left({f}_{n}{f}_{k}^{\prime}r\right)+nA(r^{\prime} )\\ 	=25.48+0.13n+0.07n+0.09n=25.48+0.29n\,\text{min.},$$

#### Simulated data for training and testing

We used ART^67^ to generate *r* = 6 × 10^8^ reads from human reference genome (30X coverage) with Illumina HiSeq 2500 error profile. For each target locus, we modified the number of the repeats to be ±3 of the original count in the reference with setting 1 as minimum number of repeats, and simulated reads from those regions. For each locus, we assigned labels to reads as being true reads or not, based on exact location. We divided the original set of reads into three parts: 70% for training, 10% for validation, and 20% for testing. We trained all neural network models using the training and validation sets, and reported performance on the test dataset.

To augment the data, we added random single nucleotide variations in the genome sequences of the dataset before simulating the sequencing reads^68^. For each sequence in the dataset, we replaced its nucleotides with a random one with probability *r*_m_. We set *r*_m_ = 10^−5^, the novel base substitution mutation rate within VNTRs^69^. This method of dataset augmentation helps include “mutated” k-mers in the embedding of reads, making the method more robust.

#### adVNTR-NN accuracy versus other methods

To test and compare genotyping accuracy against VNTRseek (v1.10.0), we started with a random selection of 10,000 target VNTR loci (<140 bp) and filtered them out if a VNTR locus was marked as indistinguishable in VNTRseek. As a result, 9638 target VNTRs remained. We used ART^67^ to generate heterozygous samples by simulating 15X coverage reads from each modified haplotype which contained a non-reference allele and combined those with 15X reads that were simulated from reference. The non-reference allele for each VNTR was chosen to be in the range [*c* − 3, *c* + 3], where *c* is the reference count. Together, this provided six diploid simulated datasets for each locus, at 30X coverage.

Similarly, to test and compare genotyping accuracy against GangSTR^40^ (v2.4.5) for, we selected VNTR loci with repeat unit length ≤20 bp. A total of 6508 target VNTRs remained. Following the method for VNTRseek comparisons, we used ART^67^ to generate a homozygous sample and six heterozygous samples by simulating 30X paired-end reads with Illumina HiSeq 2500 error profile.

#### Performance test

We measured running time of adVNTR-NN and VNTRseek by running them with default parameters on a single core of Intel Xeon CPU E5-2643 v2 3.50GHz CPU. To measure the accuracy of genotyping, we ran adVNTR-NN and VNTRseek on diploid simulated data of heterozygous VNTRs and measured the number of correct calls divided by total number of VNTR loci.

### Data and preprocessing

We accessed 30X Illumina WGS data from the GTEx cohort (652 individuals) through dbGaP (accession id phs000424.v8.p2 [https://www.ncbi.nlm.nih.gov/projects/gap/cgi-bin/study.cgi?study_id=phs000424.v8.p2]). Specifically, we accessed CRAM files containing read alignments to the GRCh38 reference genome through cloud-hosted SRA data using fusera v1.0 and downloaded VCF files containing SNP genotype calls from dbGaP.

As genotyping VNTRs remains computationally expensive, we focused on the smaller set of VNTRs located within coding, untranslated, or promoter regions of genes, which are most likely to be involved in regulation. We identified VNTRs in coding exons and UTRs by intersecting VNTR coordinates with refseq gene coordinates downloaded from UCSC Table Browser. To identify VNTRs that appear within promoter regions, we considered 500 bp upstream of the TSS of genes as the promoter regions. Overall, this procedure identified 13,081 VNTRs, of which 10,262 were within the size range for short-read genotyping (Fig. 1a). We subsequently added two VNTRs previously linked to a human disease to obtain 10,264 target loci^38,38^. We genotyped these VNTR loci in 652 individuals from GTEx cohort using adVNTR-NN on Amazon Web Services (AWS) cloud, which allowed us to do the computation in parallel for different samples.

We compared the most common allele of each VNTR with the reference allele (GRCh38) to observe representation of each VNTR in the reference. We also searched for VNTRs with multiple observed alleles to estimate a rate of polymorphism for VNTRs and find how common each allele was. To call a VNTR polymorphic, we set the MAF at 5% and any variation below that frequency was discarded. In addition, we identified the amount of base-pair difference that they make in genome of each individual by comparing the copy number difference of VNTRs between reference and the sample and multiplied that by the pattern length of each locus. We computed how many loci on average differed between an individual and reference by combining all non-reference calls in at least one haplotype from all individuals and dividing it by all called variants. VNTRs whose allele frequencies did not meet the expected percentage of homozygous versus heterozygous calls under Hardy–Weinberg equilibrium (*P* < 0.05 for two-sided binomial test) were eliminated. We further removed VNTRs that were monomorphic (only one allele) in the entire GTEx cohort or had MAF lower than 1% among the individuals with expression data in every tissue. We used the resulting 2672 VNTRs for subsequent analysis (Supplementary Data 1).

We obtained processed RNA-expression data (RPKM values) from 54 tissues from dbGaP (phs000424.v7.p2 [https://www.ncbi.nlm.nih.gov/projects/gap/cgi-bin/study.cgi?study_id=phs000424.v7.p2]) and limited analysis to 46 tissues which had data for at least 100 individuals. “Non-expressed genes”—genes with median RPKM level zero—in each tissue were removed from analysis. For the remaining genes, we quantile-normalized RPKM values of each tissue to a normal distribution. We analyzed VNTR-gene pairs for each VNTR and its closest gene based on refseq annotations in each of the 46 tissues.

### Identification of eVNTRs

Before the analysis of the association of VNTR genotypes and gene expression levels, we adjusted gene expression levels for each tissue in order to control for covariates of sex, population structure, and technical variations in measuring expression. For population structure, we used the top ten PCs from a principal components analysis (PCA) on the matrix of SNP genotypes to provide a correction for population structure. To generate the SNP genotype matrix, we used the VCF files for GTEx cohort (accession phg001219 [https://www.ncbi.nlm.nih.gov/projects/gap/cgi-bin/study.cgi?study_id=phs000424.v7.p2]) and filtered biallelic SNP sites MAF >0.05 using plink^70^. To correct for non-genetic factors such as technical variations in measuring RNA expression levels (e.g., batch effects, environmental variables), we applied PEER factor correction and used the top 15 factors^43^. We removed the effect of covariates by regressing them out from the RNA expression matrix of each tissue and subtracting their factor contributions and used the residuals for all eQTL association analyses.

We normalized the individual raw gene expression values to *N*(0, 1) by subtracting the mean and dividing by the standard deviation of the expression values for that cohort. For a gene-VNTR pair *v*, let *y*_iv_ denote the normalized expression value of gene in *v* for individual *i* and *x*_iv_ denote the genotype of the VNTR in *v* for individual *i*. Then,

6 $${y}_{iv}={\beta }_{v}{x}_{iv}+\mathop{\sum }\limits_{k}{\gamma }_{k}{\text{PC}}_{ik}+\mathop{\sum }\limits_{k}{\delta }_{k}{R}_{ik}+{\epsilon }_{iv}$$

where, PC_ik_ denotes the strength of the *k*-th principal component, and *R*_ik_ the value of the *k*-th PEER factor. We performed the association test for each VNTR-gene pair separately for each tissue type using Python statsmodels linear regression, ordinary least squares (OLS)^71^, and computed a nominal *p* value of the strength of association for each VNTR-gene pair using two-sided Fisher’s exact test.

#### Multiple testing correction

We used permutation tests and the Benjamini–Hochberg procedure to estimate a 5% FDR significance cutoff for each tissue. The significance thresholds for each of the 46 tissues ranged from 10^−3^ to 3.8 × 10^−5^ (Fig. S13). Overall, 759 significant tests were observed from total of 73,609 tests in all tissues and 163 unique VNTRs passed the significance test in at least one tissue.

We performed a similar correction for the Geuvadis cohort. Specifically, we performed 100 permutations and used a Benjamini–Hochberg procedure to control the FDR at 5%. For the Icelandic cohort, only the VNTRs that showed significant associations in GTEx were tested using unmapped reads plus reads mapped to those specific loci. Hence, we used the conservative *p* value cutoff from whole blood tissue of the smaller GTEx cohort.

#### Fine-mapping of causal variants

To compare the strength of the VNTR association relative to proximal SNPs, we extracted all SNPs from 50 kb 5′ to the transcription start, from the gene body, and up to 50 kb 3′ to the end of the transcript using the GTEx variant calls. To perform a fair comparison, we used the same test and covariates for VNTRs and repeated it for each SNP by replacing the genotype to obtain the strength of association for each SNP. Then, we ranked all variants based on their association *P* value.

We further used a fine-mapping method, CAVIAR, as an orthogonal method to identify the causal variant for the change in gene expression level. CAVIAR is a statistical method that quantifies the probability that a variant is causal by combining association signals (i.e., summary level Z-scores) and LD structure between every pair of variants^47^. We ran CAVIAR with parameter -c 1 to identify the most likely causal variant, along with the causality probability distribution for each variant site. We ranked variants based on their causality probability given by CAVIAR and called it the causality rank.

### Reporting summary

Further information on research design is available in the Nature Research Reporting Summary linked to this article.

## Supplementary information

Supplementary Information

Description of Additional Supplementary Files

Supplementary Data 1

Supplementary Data 2

Supplementary Data 3

Supplementary Data 4

Supplementary Data 5

Supplementary Software 1

Supplementary Software 2

Reporting Summary

## Data Availability

The analyses presented in this paper are based on the use of GTEx study data downloaded from the dbGaP web site, under phs000424.v7.p2 [https://www.ncbi.nlm.nih.gov/projects/gap/cgi-bin/study.cgi?study_id=phs000424.v7.p2]. The 30X whole genome sequencing data of 1000 Genomes Project samples used in this research were generated at the New York Genome Center with funds provided by NHGRI Grant 3UM1HG008901-03S1. This sequencing data is available at ENA Study PRJEB31736 [https://www.ebi.ac.uk/ena/browser/view/PRJEB31736] and ENA study PRJEB36890 [https://www.ebi.ac.uk/ena/browser/view/PRJEB36890]. RNA-seq data corresponding to 465 samples from 1000 Genomes Project were downloaded from Geuvadis project [https://www.ebi.ac.uk/arrayexpress/experiments/E-GEUV-1/]. The refseq data is vailable at UCSC Table Browser [https://genome.ucsc.edu/cgi-bin/hgTables]. Source data are provided with this paper.

## Code availability

adVNTR-NN is available at https://github.com/mehrdadbakhtiari/adVNTR. v1.4.0 of the software was used for this paper (Supplementary Software 1). Code for eVNTR analysis and generating figures is available at https://github.com/mehrdadbakhtiari/VNTR-eQTL and as Supplementary Software 2^72^.
