# Supplementary material for: Variable number tandem repeats mediate the expression of proximal genes
Source: Nat Commun. 2021 Apr 6;12:2075. doi: 10.1038/s41467-021-22206-z (PMC8024321; doi:10.1038/s41467-021-22206-z)
Supplement: Supplementary file 1 — Supplementary Information [file 41467_2021_22206_MOESM1_ESM.pdf]

Supplementary Information for

Variable Number Tandem Repeats mediate the expression of proximal genes

Bakhtiari *et al.*

Supplementary Figures

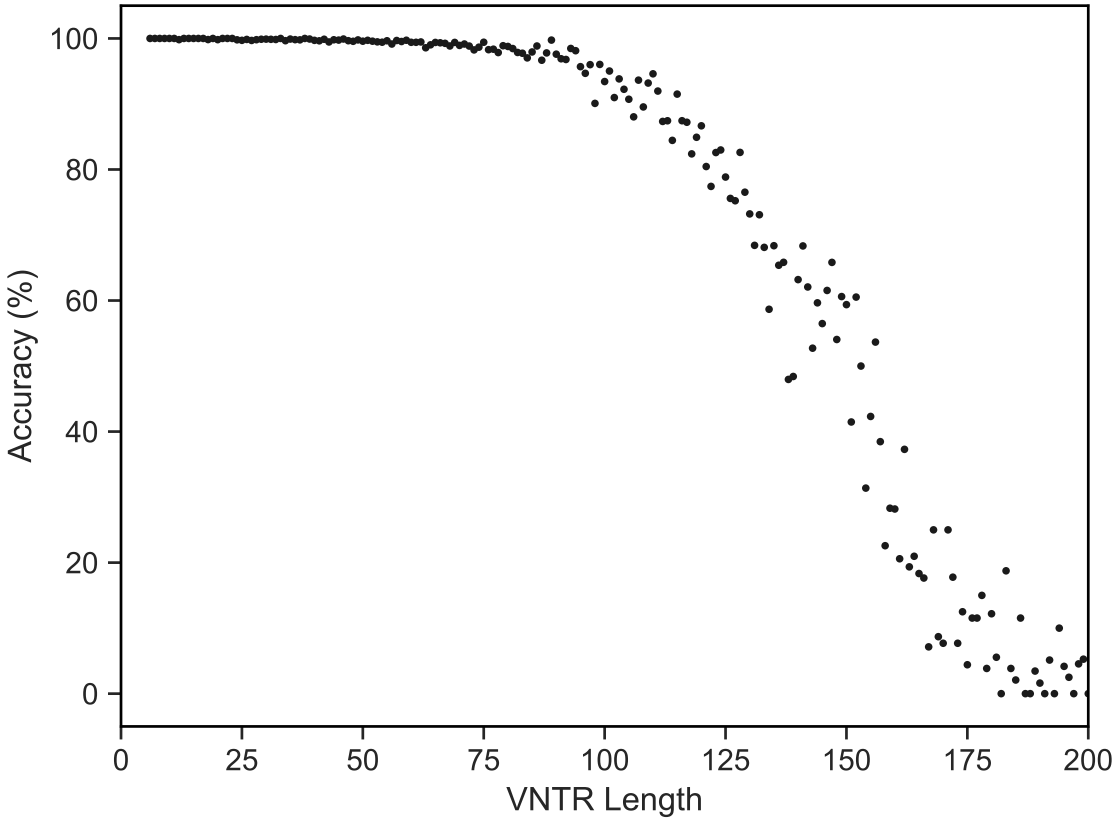

Figure S1: **Distribution of genotyping accuracy of adVNTR-NN stratified by VNTR length on simulated VNTRs.** Heterozygous alleles were simulated by inserting or deleting repeating units in one reference allele to transform its RU count c to c+x, where c is the hg19 reference count, and $x \in [-3, 3]$. Source data are provided as a Source Data file.

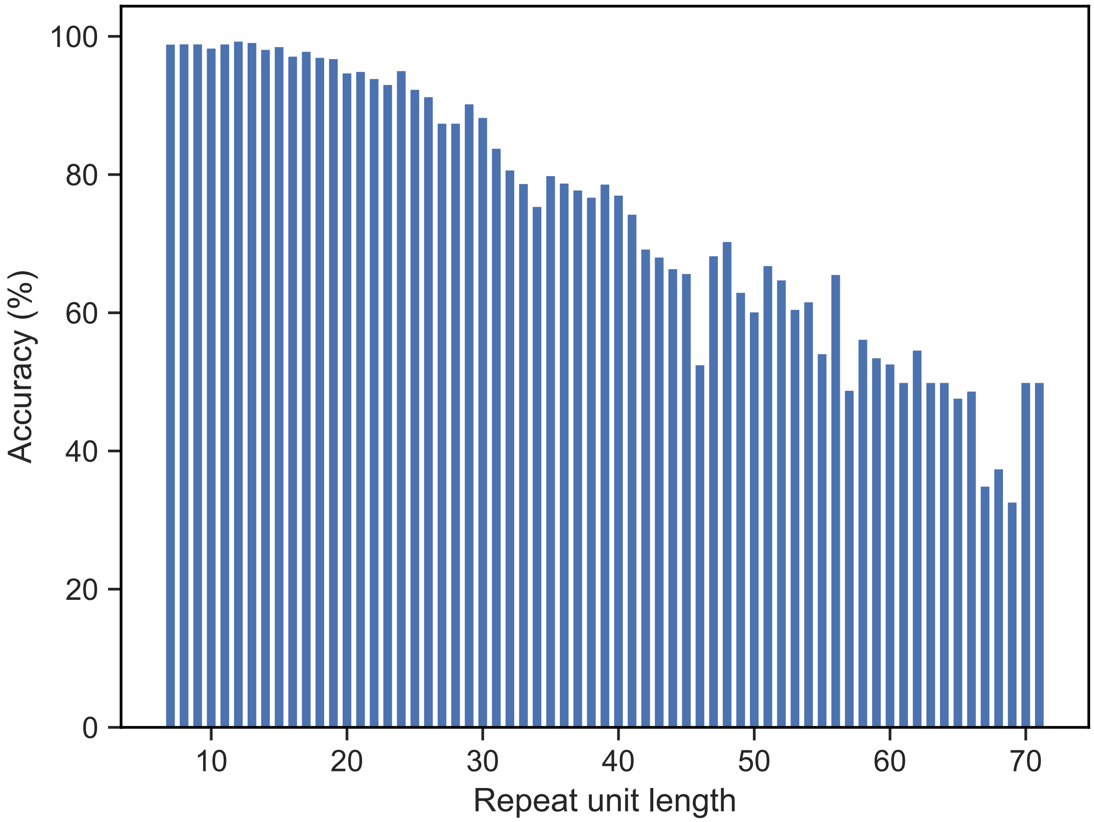

Figure S2: **Distribution of genotyping accuracy of adVNTR-NN stratified by repeat length for simulated heterozygous reads.** Heterozygous alleles were simulated by inserting or deleting repeating units in one reference allele to transform its RU count c to c+x, where c is the hg19 reference count, and $x \in [-3, 3]$. Source data are provided as a Source Data file.

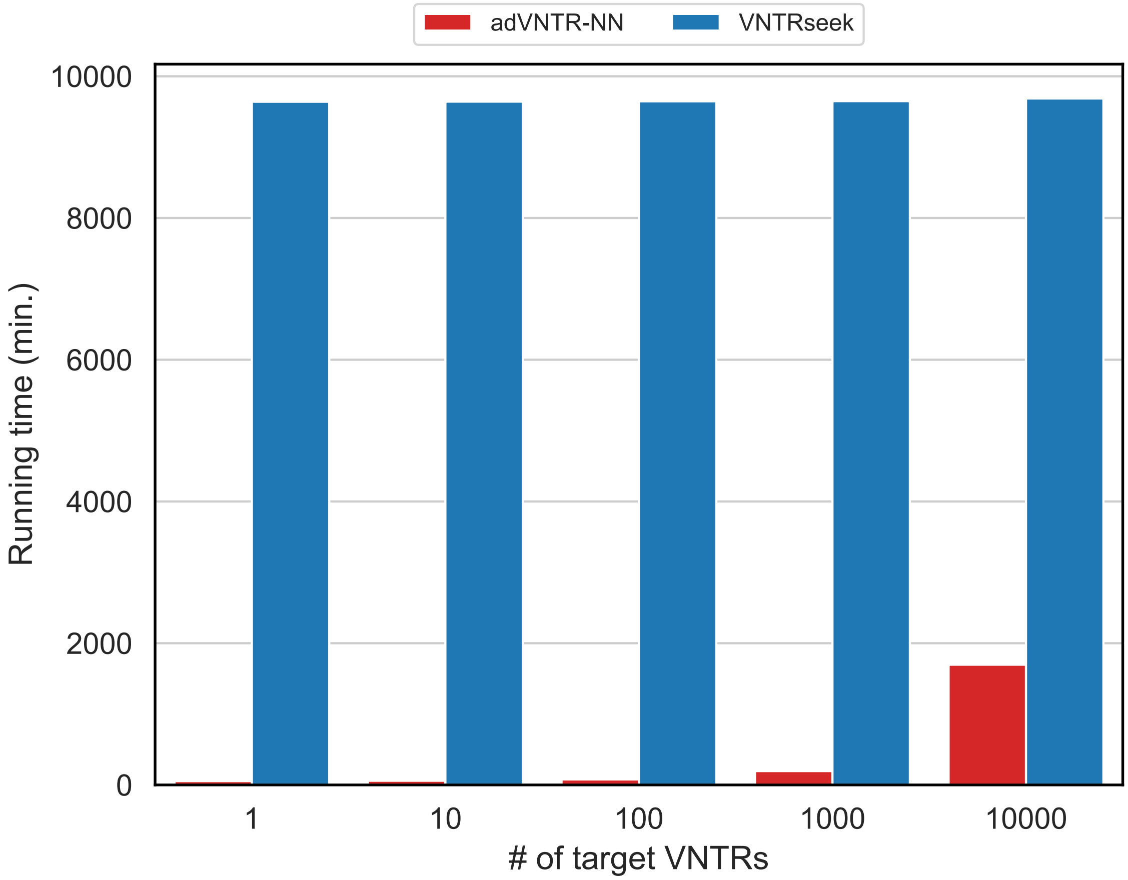

Figure S3: **Running time comparison.** Running time comparison on 1, 10, 100, 1,000, and 10,000 VNTR loci of one individual (NA24149) with 1.16×10^9 reads. Source data are provided as a Source Data file.

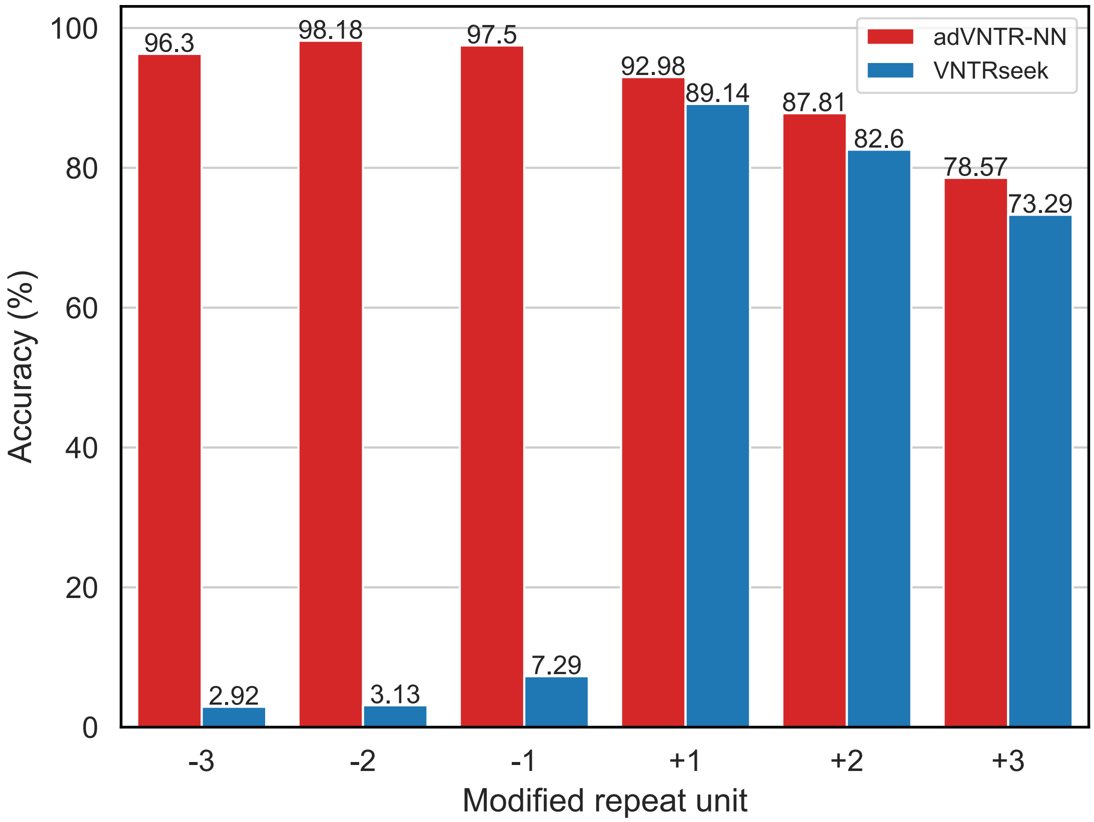

Figure S4: **adVNTR-NN and VNTRseek genotyping accuracy on simulated heterozygous reads.** The genotyping accuracy for each scenario is defined by the the number of VNTR loci correctly genotyped correctly divided by the number of VNTR loci. Six different heterozygous VNTR scenarios were tested; specifically, c/c-3, c/c-2, c/c-1, c/c+1, c/c+2, c/c+3, where c is the hg19 reference count. The number of VNTR loci modified for contraction scenarios were 9,638 (c-1), 5,078 (c-2), and 2,084 (c-3), with the reductions happening due to a requirement of at least 1 repeating copy for each VNTR allele. All expansion scenarios had 9,638 VNTRs. Source data are provided as a Source Data file.

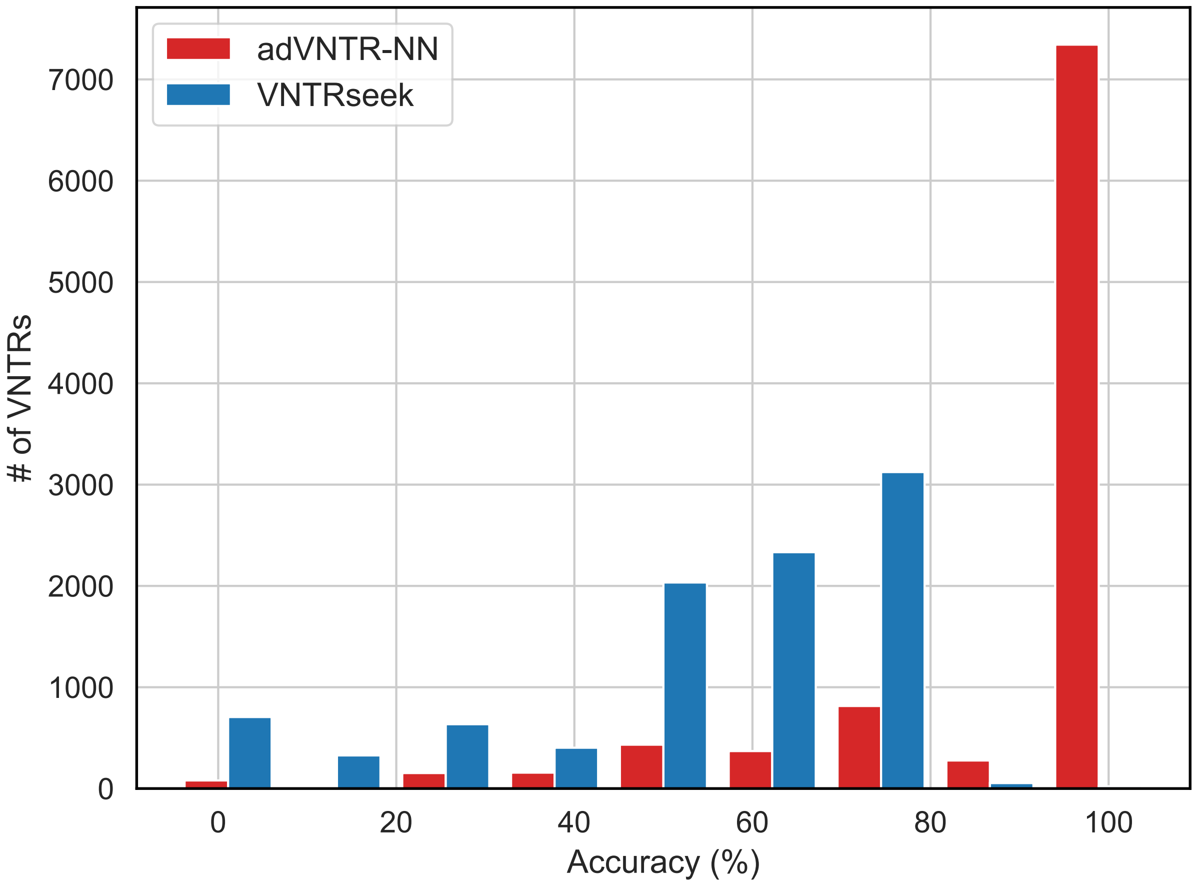

Figure S5: **Distribution of genotyping accuracy of adVNTR-NN and VNTRseek on simulated heterozygous reads.** The genotyping accuracy for each VNTR is defined by the number of loci genotyped correctly divided by the number of loci. Six different heterozygous VNTR scenarios were tested; specifically, c/c-3, c/c-2, c/c-1, c/c+1, c/c+2, c/c+3, where c is the hg19 reference count. The number of VNTR loci modified for contraction scenarios were 9,638 (c-1), 5,078 (c-2), and 2,084 (c-3), with the reductions happening due to a requirement of at least 1 repeating copy for each VNTR allele. All expansion scenarios had 9,638 VNTRs. adVNTR-NN had 100% accuracy in 7,343 (76%) of 9,638 VNTRs. Source data are provided as a Source Data file.

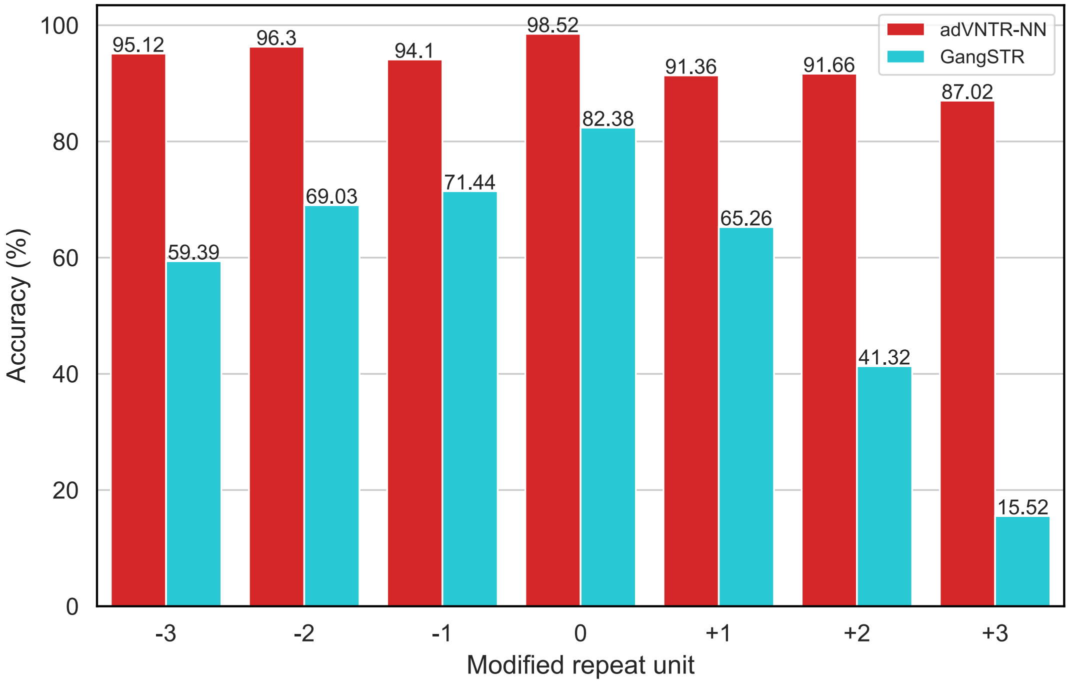

Figure S6: **adVNTR-NN versus GangSTR accuracy on simulated heterozygous reads for short RU lengths ($\leq$ 20).** Seven scenarios were tested; specifically, c/c-3, c/c-2, c/c, c/c-1, c/c+1, c/c+2, c/c+3, where c is the hg38 reference count. The genotyping accuracy for each scenario is defined by the number of VNTR loci genotyped correctly divided by the number of VNTR loci. The number of VNTR loci modified for contraction scenarios were 6,508 (c-1), 4,763 (c-2), and 2,805 (c-3), with the reductions happening due to a requirement of at least 1 repeating copy for each VNTR allele. All expansion scenarios and the homozygous case had 6,508 VNTRs. Source data are provided as a Source Data file.

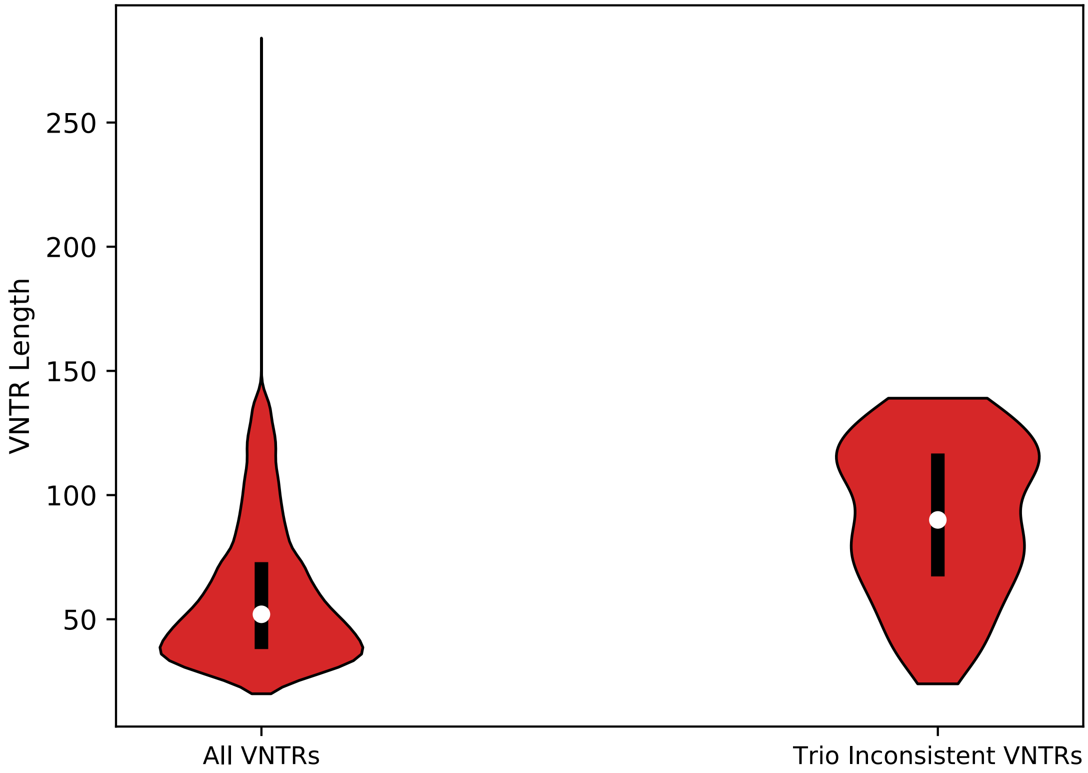

Figure S7: **Length distribution of VNTRs.** The violin plots show the distribution of VNTR lengths in 537 Trios from the 1000 Genomes Project (n=10,264 VNTRs for each trio). White dots show median values and boxes span from the 25th percentile (Q1) to the 75th percentile (Q3). VNTRs that showed consistency with Mendelian inheritance patterns had a median length of 52bp, while inconsistent calls have a median of 90bp. Source data are provided as a Source Data file.

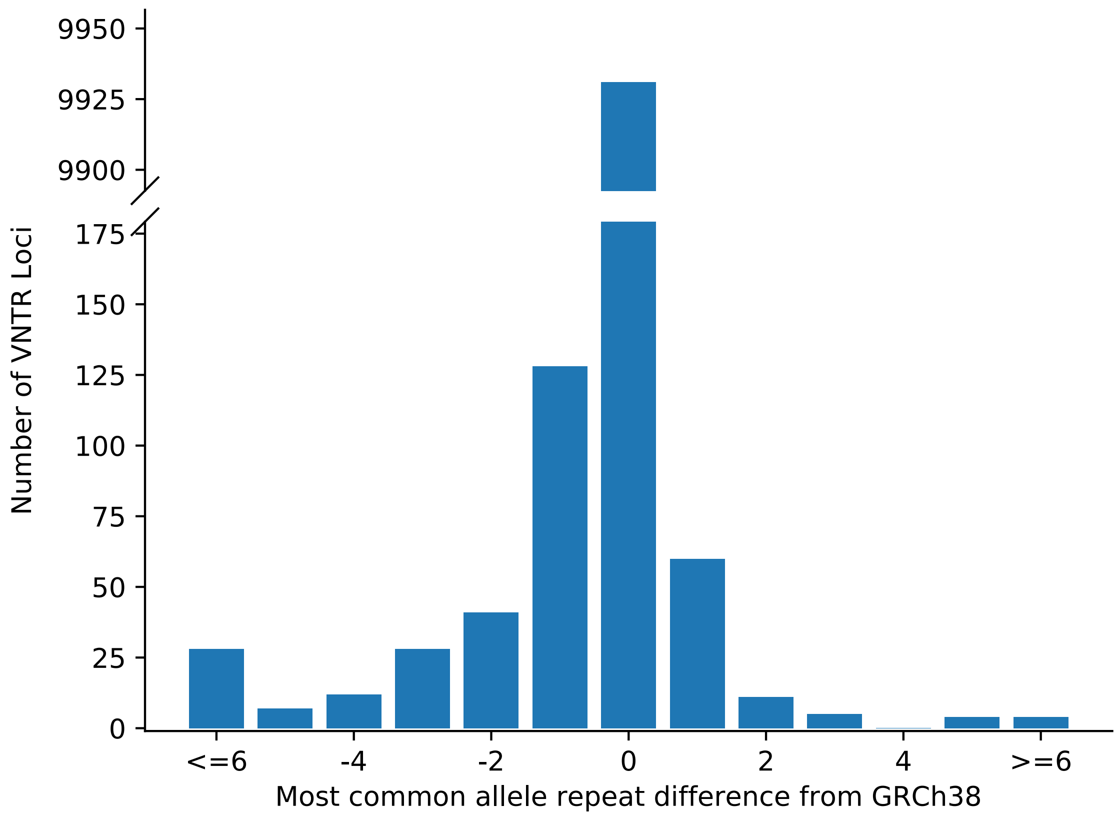

Figure S8: **Difference in VNTR loci between donors and GRCh38.** For each VNTR, the difference between the most common allele in the GTEx cohort and the GRCh38 reference repeat count was recorded. The plot shows the distribution of the differences. Source data are provided as a Source Data file.

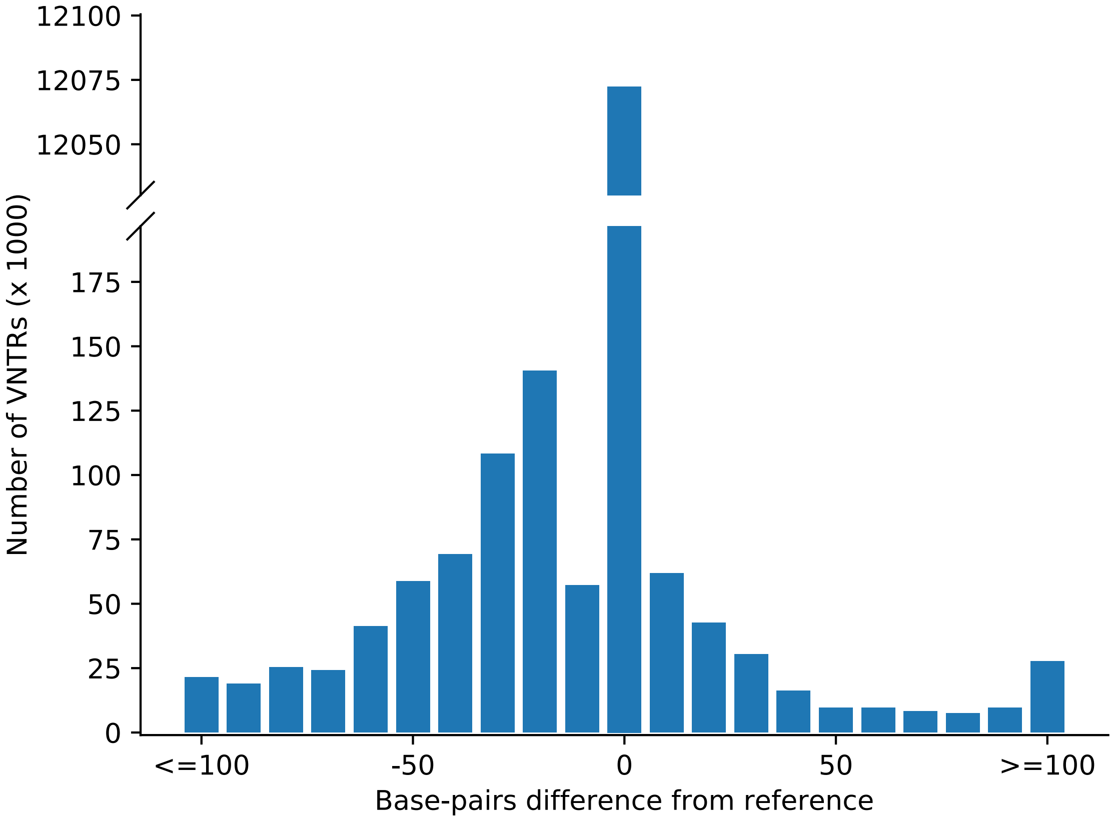

Figure S9: **Difference in VNTR loci between donors and GRCh38.** For each VNTR and each individual allele in a GTEx donor, the difference in length from the GRCh38 reference VNTR length was recorded. The plot shows a distribution of differences. Source data are provided as a Source Data file.

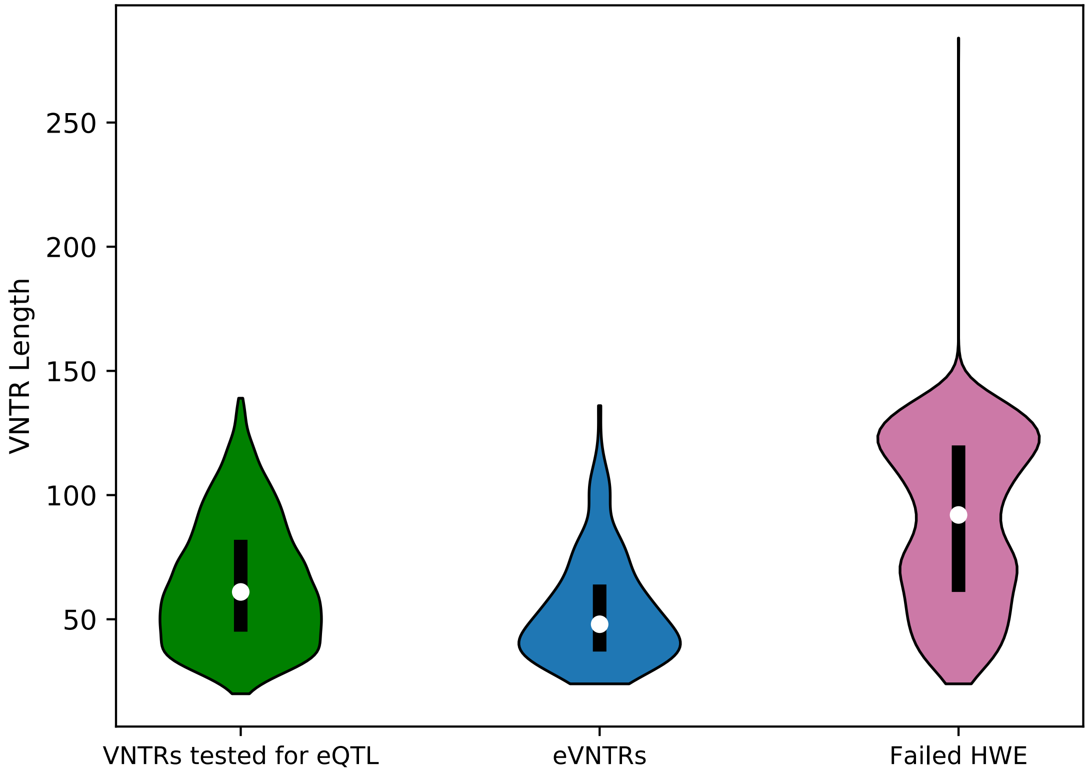

Figure S10: **Length distribution of VNTRs in the GTEx cohort (n=4,280 VNTRs).** White dots show median values and boxes span from the 25th percentile (Q1) to the 75th percentile (Q3). The length distribution for all VNTRs that passed filters had a median of 61, slightly larger than eVNTRs (median length: 48bp). In contrast, the VNTRs that failed the HWE test had a length distribution (median: 92bp), which was similar to VNTRs that showed inconsistent Mendelian inheritance patterns in trios from the 1000 Genome data (median: 90 bp; Fig. S7). Source data are provided as a Source Data file.

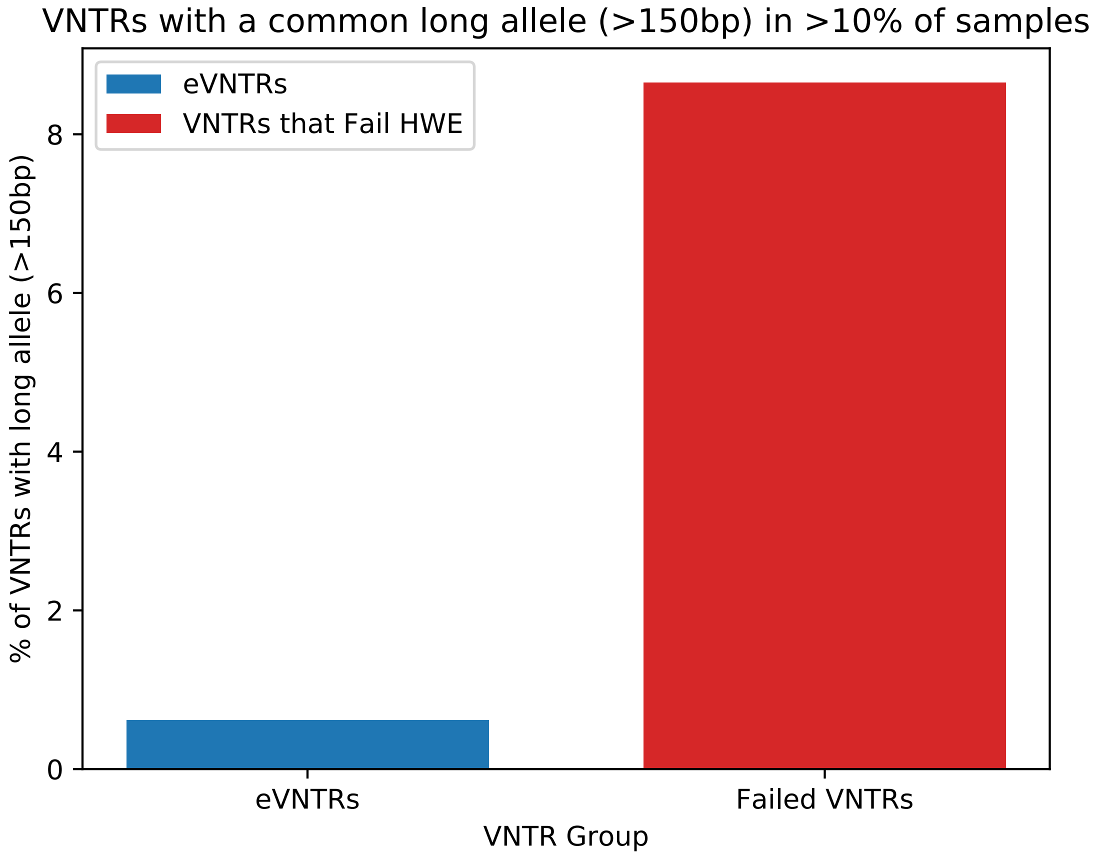

Figure S11: **Fraction of VNTRs with a common long allele.** Only one (0.6%) of the eVNTRs had a common allele (present in $> 10\%$ of samples) that was longer than a read length, while (125) 8.06% of VNTRs that fail HWE test had a common long allele. Shorter alleles are genotyped more accurately. Source data are provided as a Source Data file.

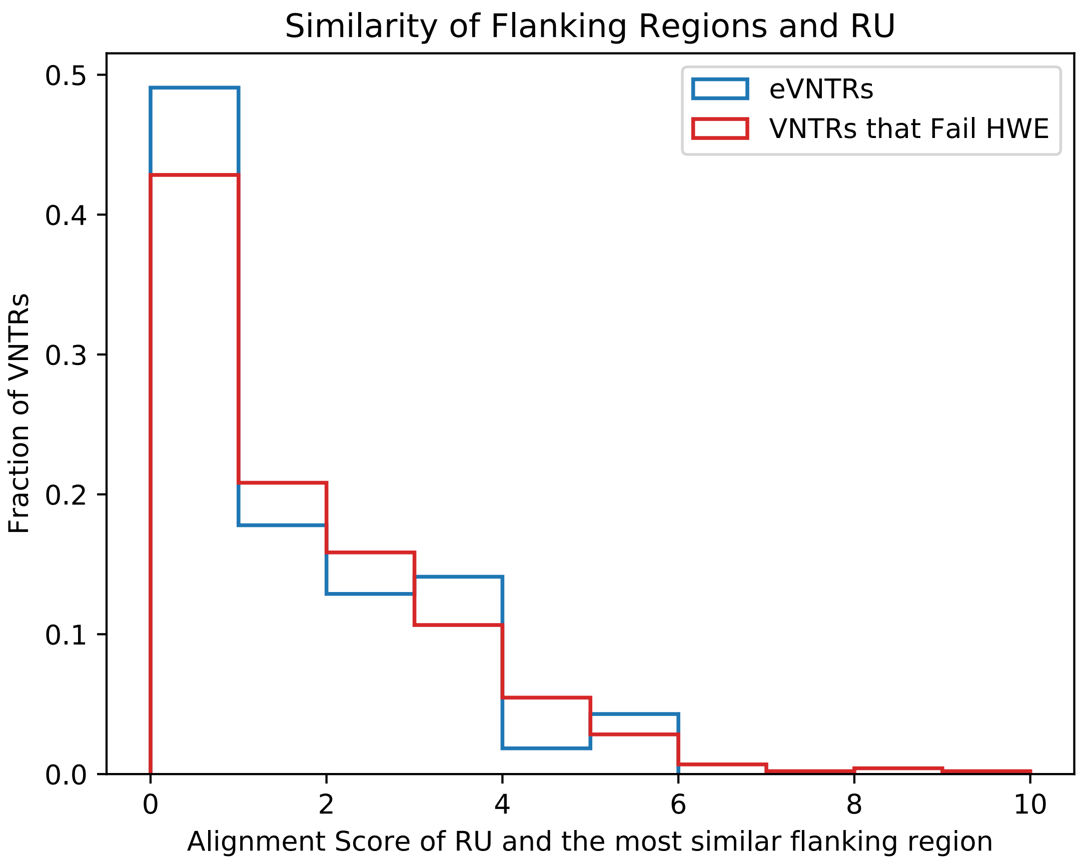

Figure S12: **Similarity of VNTR repeating pattern with flanking region.** Distribution of the number of bases in the repeat unit of VNTRs that identically match a flanking region. Higher similarity of repeating unit and flanking region makes it more challenging to distinguish the VNTR boundary and make an accurate genotype call. Source data are provided as a Source Data file.

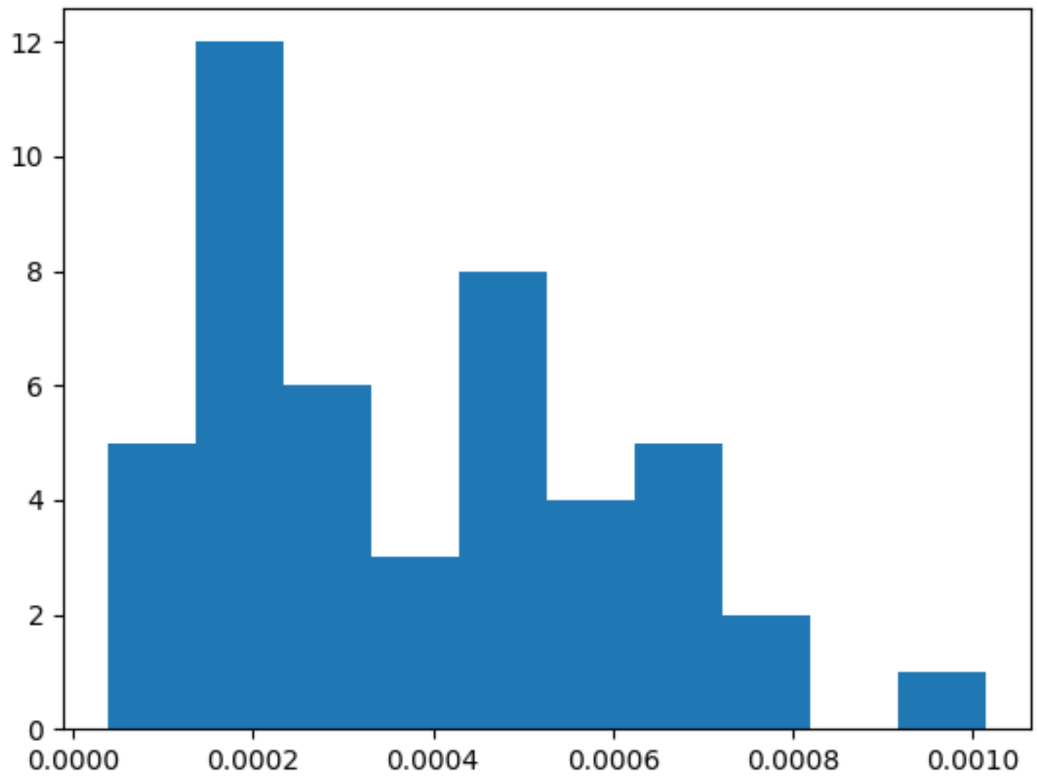

Figure S13: **Distribution of significance thresholds for association test.** Significance thresholds for each of the 46 tissues. Source data are provided as a Source Data file.

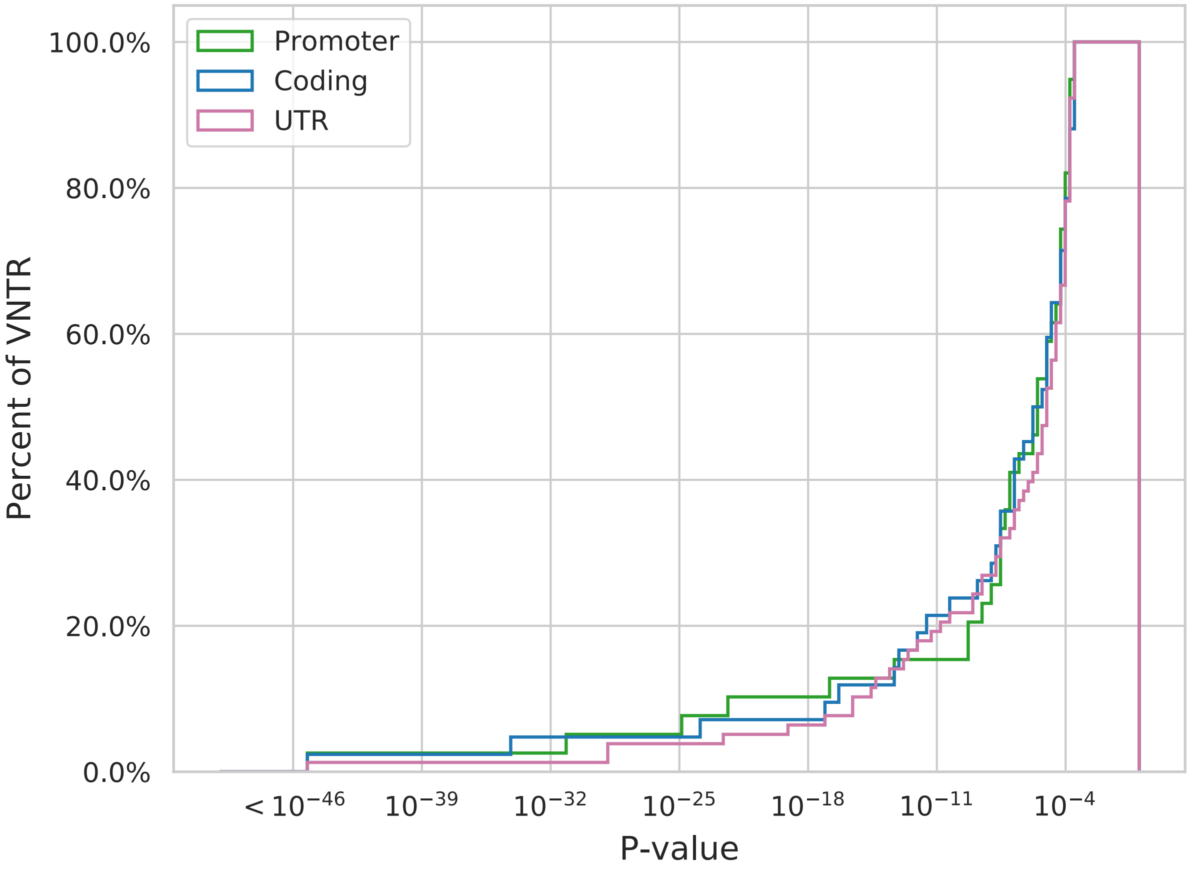

Figure S14: **Cumulative distribution of eVNTR p-values for different classes.** The plots suggest that the relative location of a genic VNTR does not significantly change the strength of association with gene expression. Source data are provided as a Source Data file.

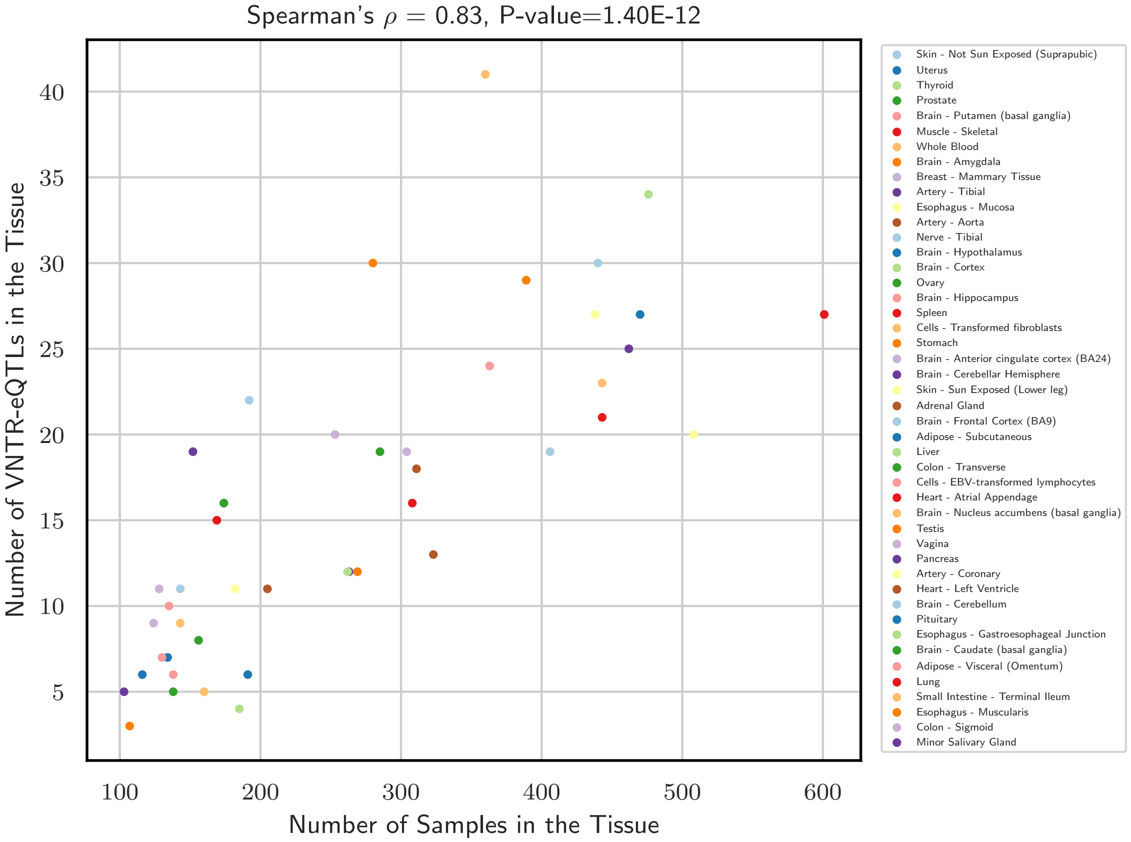

Figure S15: **Correlation between number of eVNTRs and sample-size.** Overall, we see a strong correlation between the number of samples and eVNTRs. Testis and transformed-fibroblasts had relatively higher number of eVNTRs, while fewer eVNTRs were identified in Whole blood and Skeletal muscle, relative to the sample size. Source data are provided as a Source Data file.

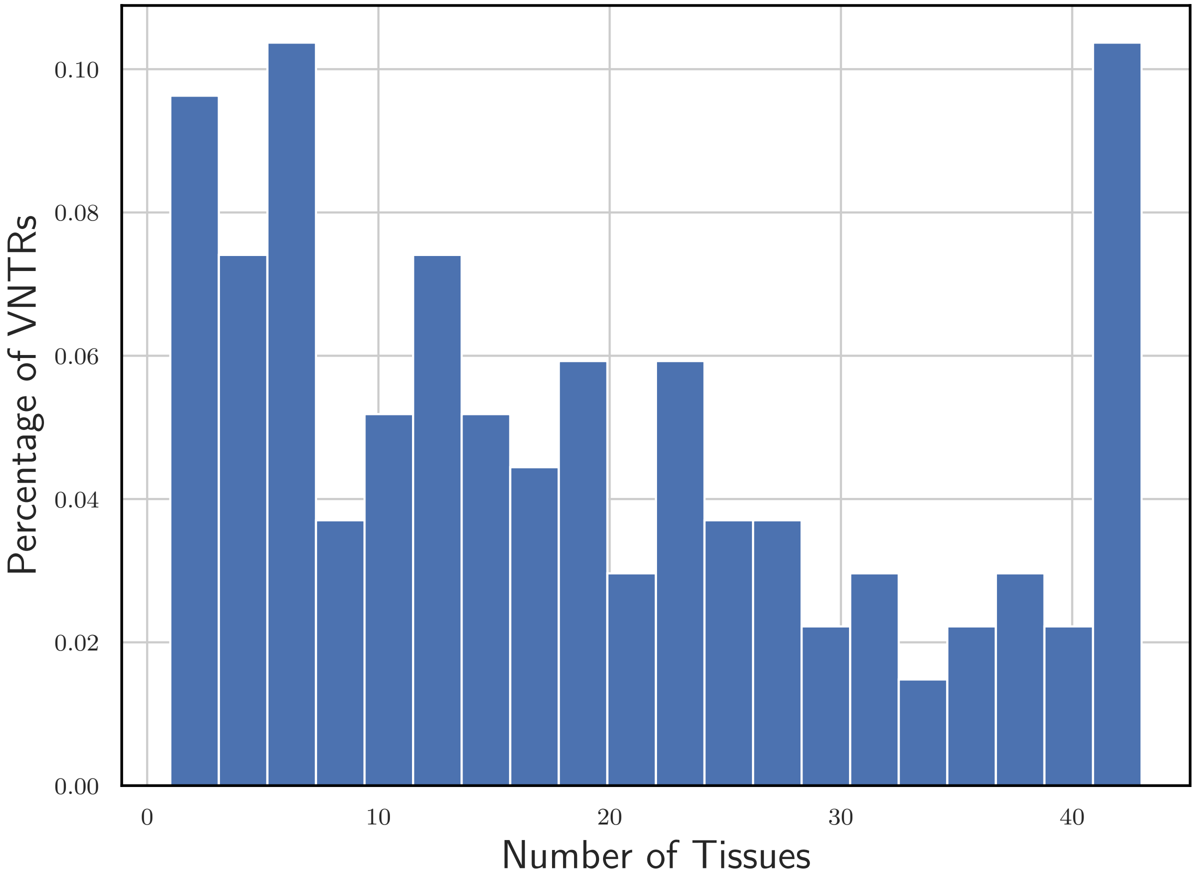

Figure S16: **Tissue sharing of eVNTRs.** The fraction of eVNTRs that are active in a specific number of tissues as determined by mash. 38% of eVNTRs were significant in at least half (23) of all tissues. Source data are provided as a Source Data file.

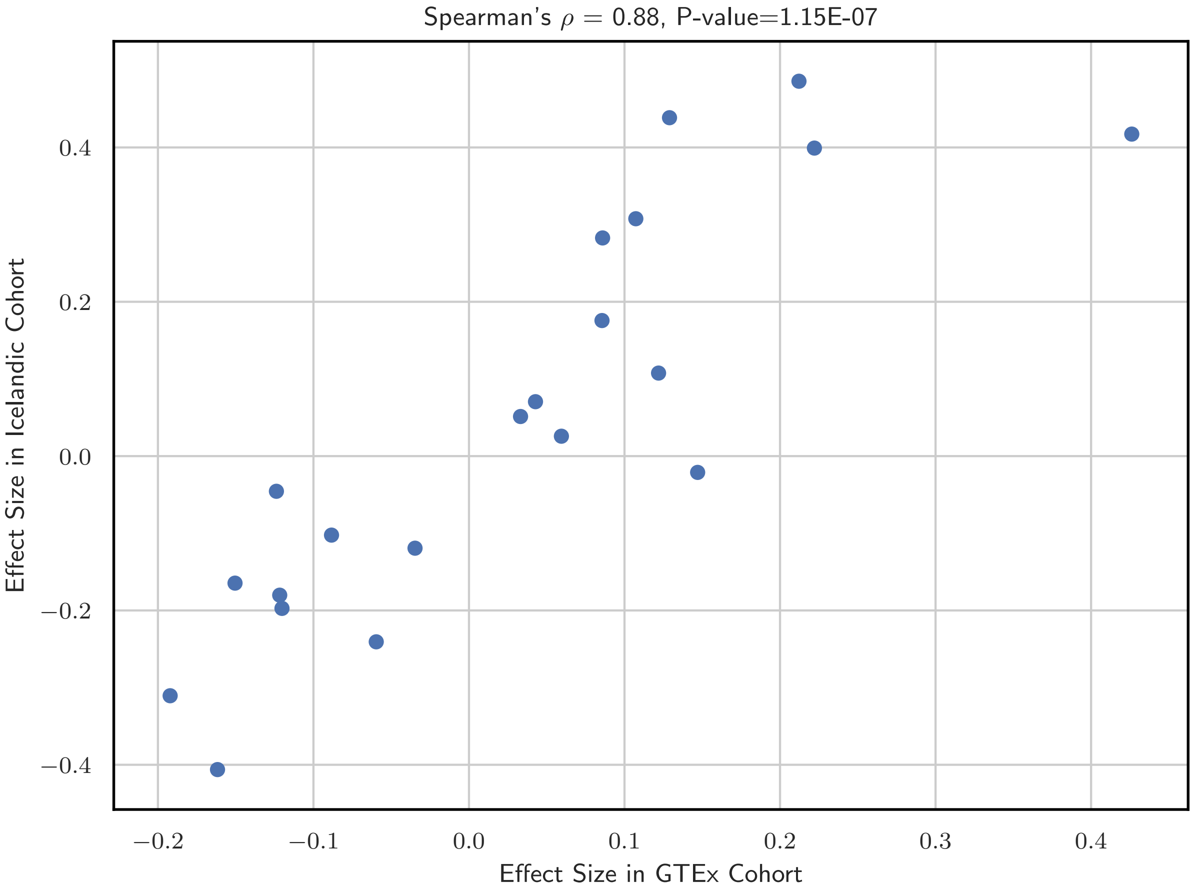

Figure S17: **Reproducibility of effect sizes in Icelandic Cohort.** The scatter plot compares the effect sizes of each eVNTR association in the GTEx cohort (x-axis) against the Icelandic cohort (y-axis) for blood tissue. The Spearman's correlation coefficient was 0.88 (p-val: $1.15E{-}07$). Source data are provided as a Source Data file.

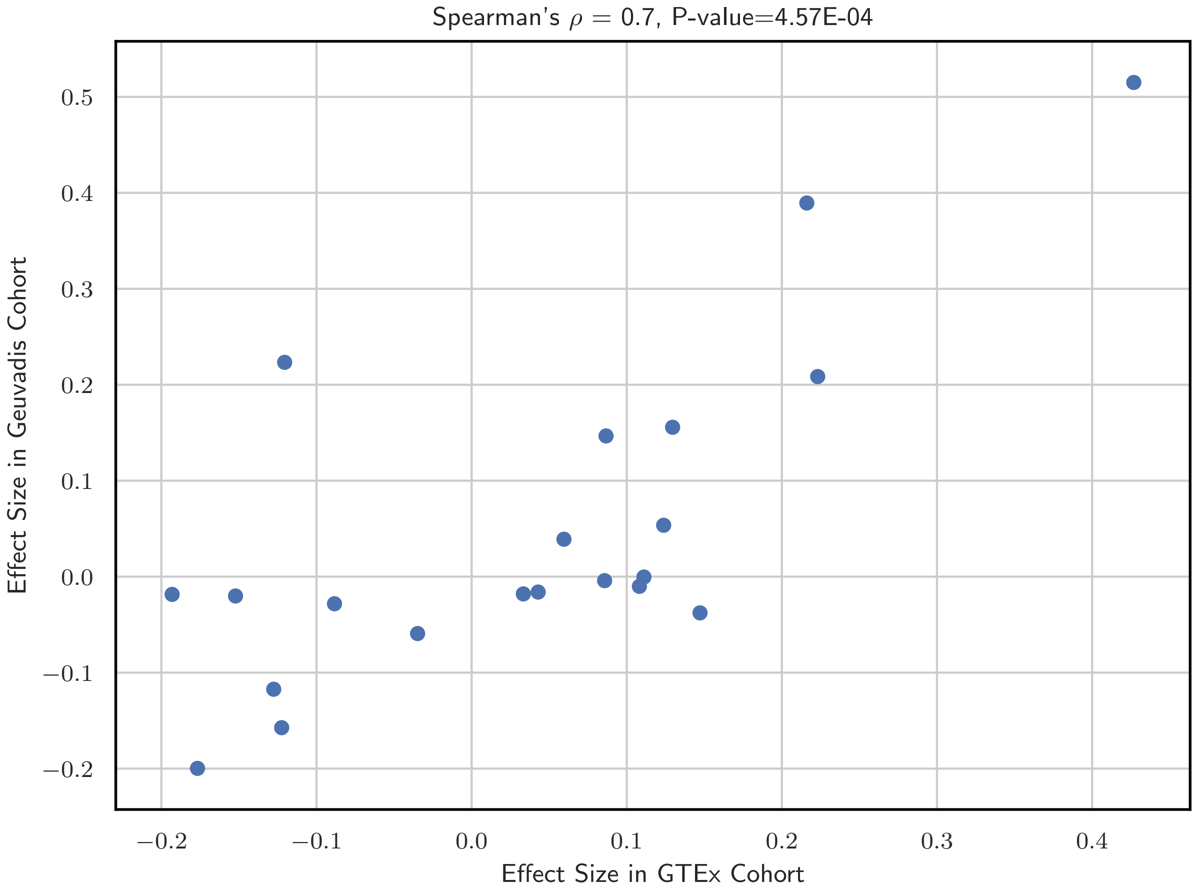

Figure S18: **Reproducibility of effect sizes in the Geuvadis Cohort.** The scatter plot compares the effect sizes of each eVNTR associations in GTEx cohort (x-axis) against the Geuvadis cohort (y-axis) for blood tissue. The Spearman's correlation coefficient was 0.7 (p-val: $4.57E-04$). Source data are provided as a Source Data file.

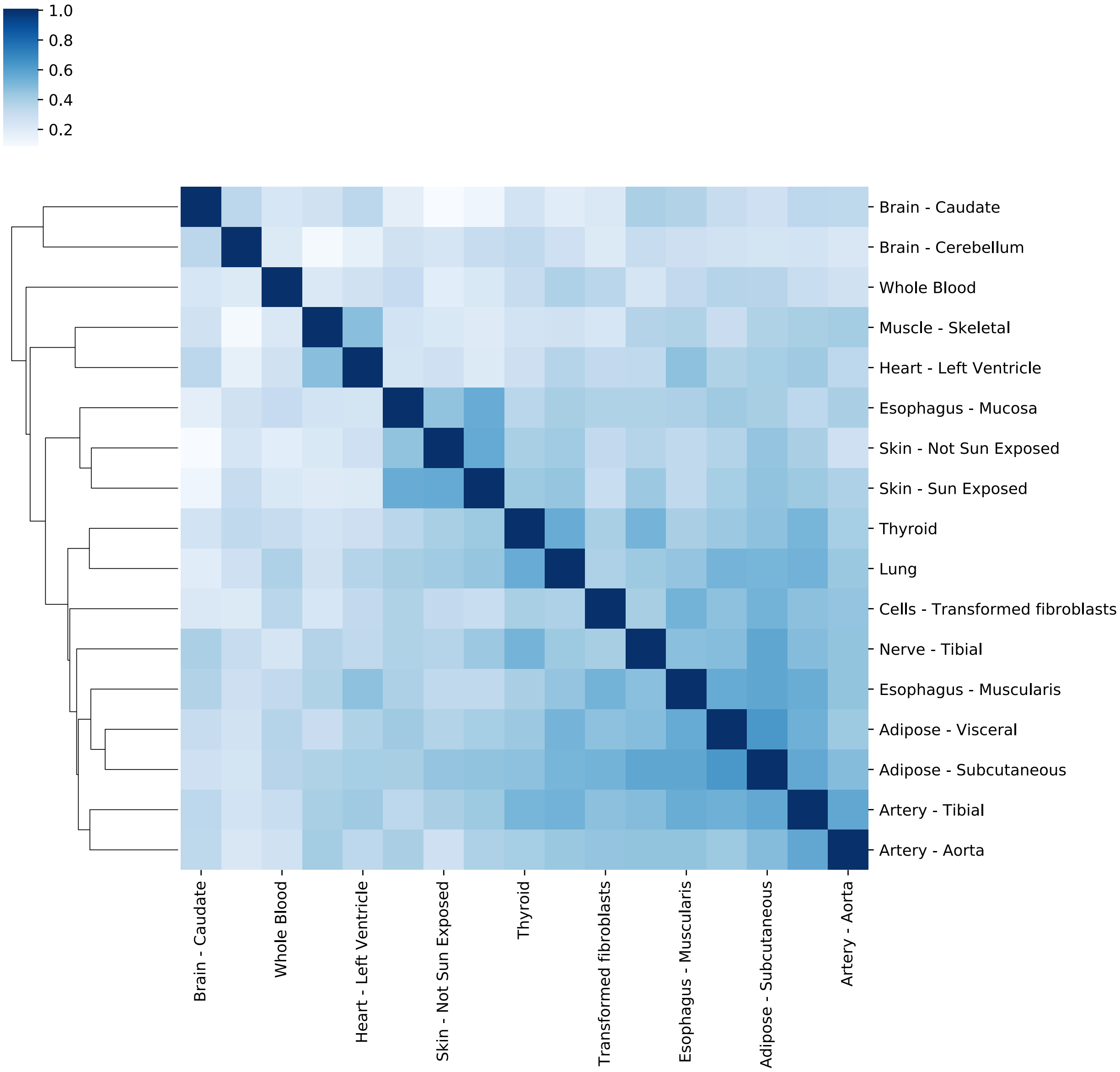

Figure S19: **Spearman correlation of eVNTRs effect sizes for pairs of tissues.** The correlation was restricted to the subset of 17 tissue types used in Fotsing[1], Fig. 1d for comparison. Source data are provided as a Source Data file.

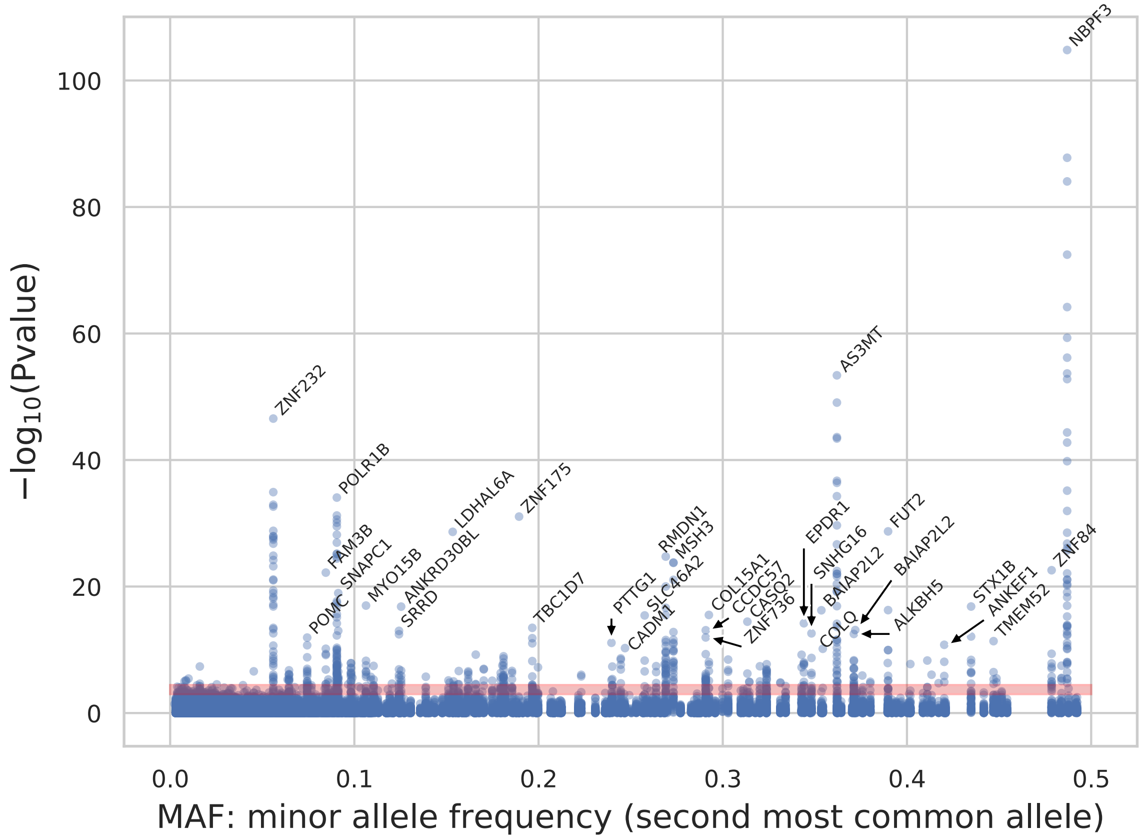

Figure S20: Significance of VNTR association with gene expression plotted against Minor Allele Frequency. The shaded region represents tissue specific false discovery rate cut-offs. Note that all significant tests for a single VNTR appear in a single column. Source data are provided as a Source Data file.

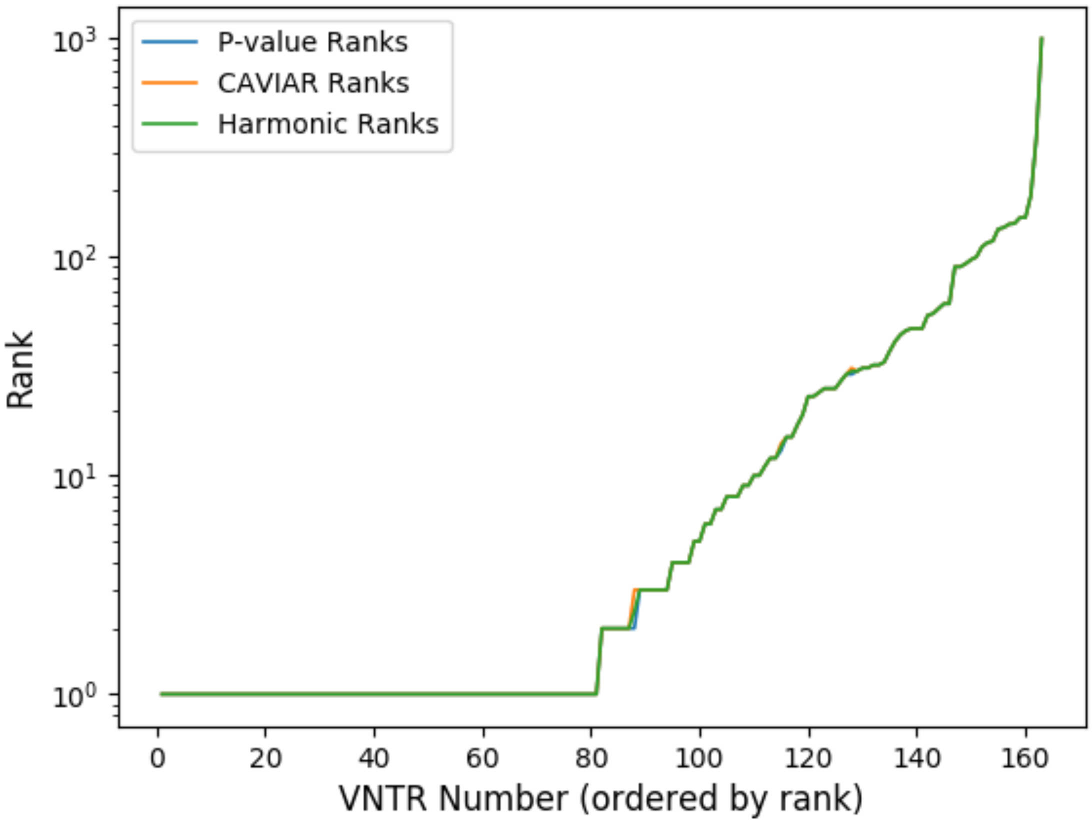

Figure S21: Causality rank of eVNTRs measured using strength of association (blue), CAVIAR (red), and mean harmonic rank (green). The P-value and CAVIAR based ranks coincide. Source data are provided as a Source Data file.

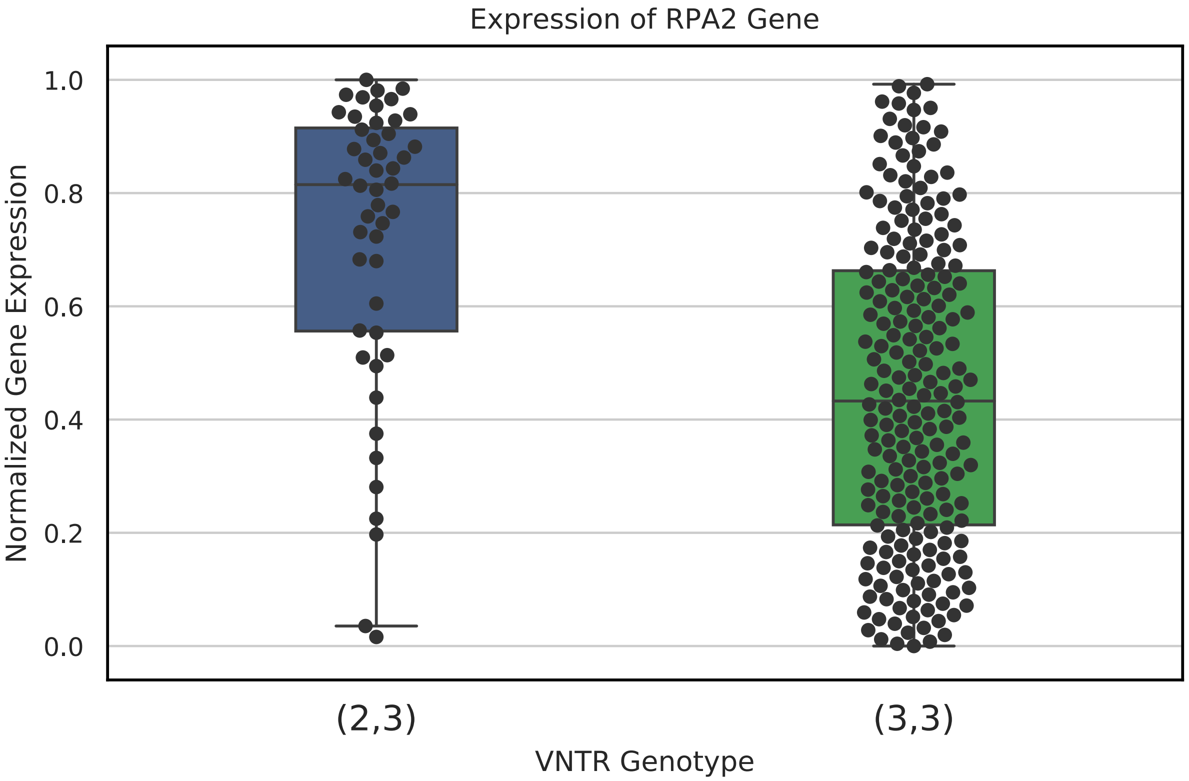

Figure S22: **Association of RPA2 VNTR genotype with gene expression level. n=254 samples, P-value** 3.79×10^{-25}. Increase RPA2 expression has been associated with worse survival outcomes in colon cancer[2]. Only two samples had a homozygous (2, 2) genotype. Their normalized expression levels were 0.85 and 0.99, which is consistent with the trend. However, they were excluded from analysis due to the small counts. Horizontal lines show median values, boxes span from the 25th percentile (Q1) to the 75th percentile (Q3). Whiskers extend to $Q1 - 1.5 \times IQR$ (bottom) and $Q3 + 1.5 \times IQR$ (top), where IQR is the interquartile range ($Q3 - Q1$). Source data are provided as a Source Data file.

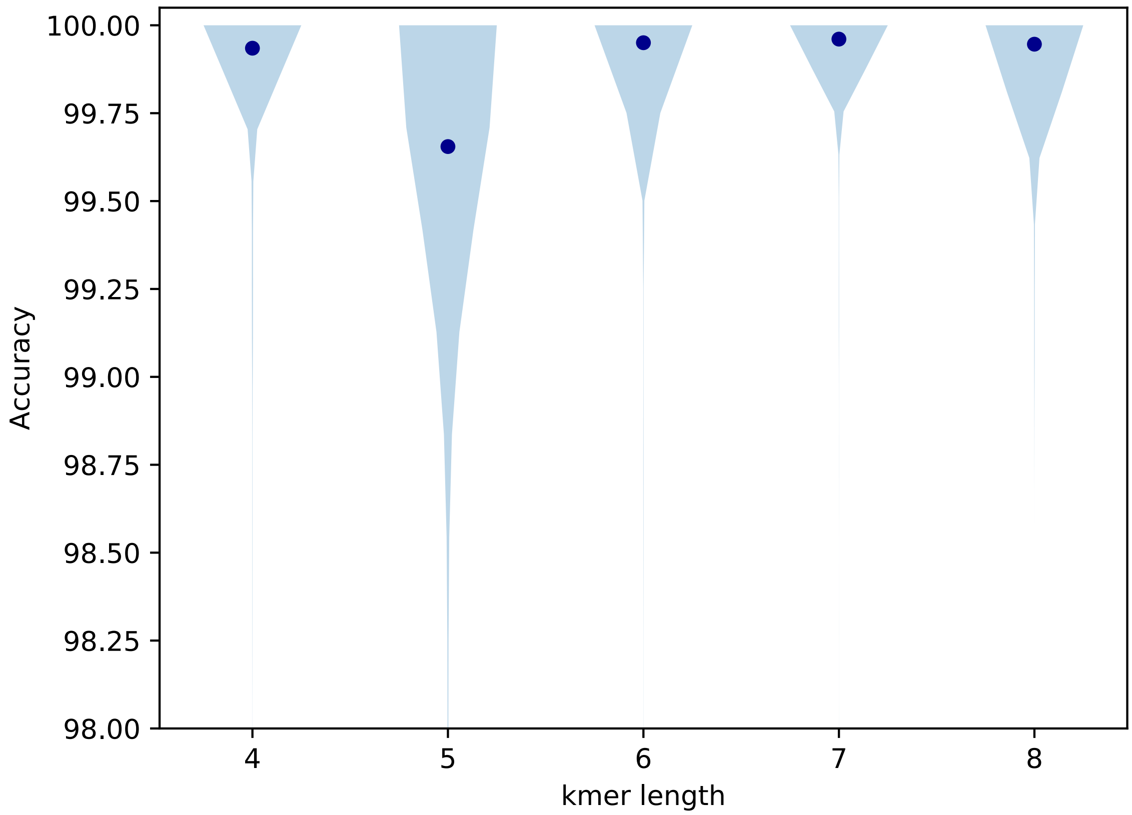

Figure S23: **Effect of kmer length on accuracy.** Performance of the neural network model on validation set for different k-mer lengths. k=6 was used for all test runs as it had the highest mean accuracy of 99.95%. Source data are provided as a Source Data file.

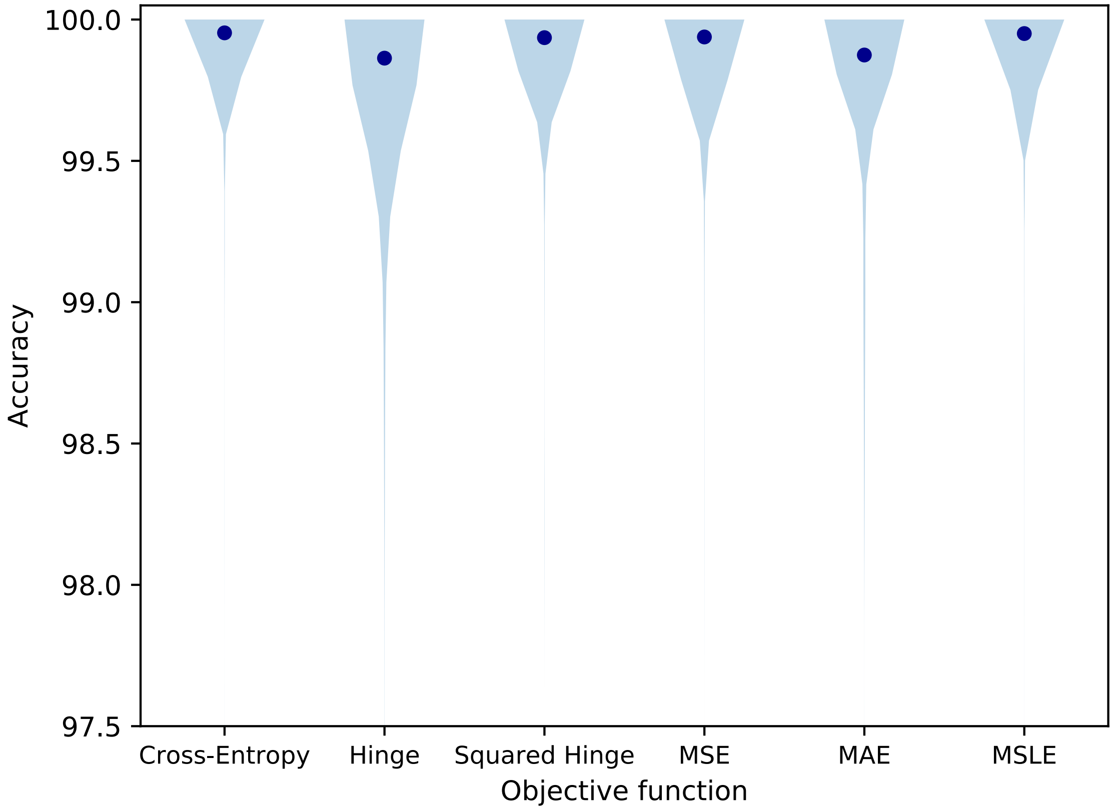

Figure S24: **Effect of loss function on accuracy.** Performance of the neural network model on validation set for different loss functions. The mean of each distribution is shown by a blue dot. Binary cross-entropy was used as the loss function for all tests. Source data are provided as a Source Data file.

Supplementary Tables

	Locus	adVNTR P-value	HipSTR P-value[1]	Replication eVNTR	Replication eSTR
1	chr6:13328502-13328532	3.39×10^{-14}	7.89×10^{-13}	Y	Y
2	chr5:160421950-160422000	7.86×10^{-12}	8.59×10^{-11}	Y	Y
3	chr22:37510301-37510338	2.29×10^{-9}	5.39×10^{-7}	Y	Y
4	chr17:63703959-63703989	4.14×10^{-7}	9.17×10^{-4}	Y	N
5	chr10:70132751-70132793	2.25×10^{-5}	3.74×10^{-3}	Y	N
6	chr11:6390700-6390749	5.80×10^{-5}	2.22×10^{-5}	Y	Y
7	chr20:35652812-35652848	1.12×10^{-4}	3.99×10^{-2}	Y	N
8	chr6:148343091-148343168	1.80×10^{-4}	3.78×10^{-3}	Y	N
9	chr22:37805258-37805313	2.12×10^{-4}	4.82×10^{-10}	Y	Y
10	chr3:51993818-51993872	2.79×10^{-4}	9.70×10^{-9}	Y	Y
11	chr16:71922603-71922638	7.05×10^{-4}	4.91×10^{-5}	N	Y
12	chr13:113119135-113119222	5.27×10^{-3}	9.45×10^{-8}	N	Y
13	chr16:67416367-67416422	9.93×10^{-3}	7.44×10^{-5}	N	Y
14	chr1:151511435-151511510	1.56×10^{-2}	6.37×10^{-5}	N	Y
15	chr11:8964363-8964423	1.56×10^{-2}	1.55×10^{-6}	N	Y

Table S1: **Comparison of hexamer eVNTRs using differing methods.** Each row describes a 6-bp variant identified either as eVNTR here or as an eSTR in Fotsing[1]. Fotsing et al. identified eSTRs with false discovery rate (FDR) $< 10\%$ in contrast to our cut-off of 5% FDR. Therefore, the nominal p-value of each association is presented for easier comparison. Despite differing methodologies and versions of GTEx, the loci are at least nominally significant ($p < 0.05$) in both tests.

References

[1] Fotsing, S. F. *et al.* The impact of short tandem repeat variation on gene expression. *Nature genetics* **51**, 1652–1659 (2019).

[2] Givalos, N. *et al.* Replication protein A is an independent prognostic indicator with potential therapeutic implications in colon cancer. *Modern Pathology* (2007).
