# Supplementary material for: Variable number tandem repeats mediate the expression of proximal genes
Source: Nat Commun. 2021 Apr 6;12:2075. doi: 10.1038/s41467-021-22206-z (PMC8024321; doi:10.1038/s41467-021-22206-z)
Supplement: Supplementary file 2 — Description of Additional Supplementary Files [file 41467_2021_22206_MOESM2_ESM.pdf]

Description of Additional Supplementary Files

File Name: Supplementary Data 1.
Description: List of 10,264 target VNTR loci used in this study.

File Name: Supplementary Data 2.
Description: Genotype discrepancy of adVNTR-NN and GangSTR on reference data.

File Name: Supplementary Data 3.
Description: Genotype discrepancy of GangSTR on reference data.

File Name: Supplementary Data 4.
Description: Genotype discrepancy of adVNTR and adVNTR-NN on target VNTR loci.

File Name: Supplementary Data 5.
Description: eVNTRs with known phenotypes. To select this smaller list, the complete list of eVN TRs (Supplementary Data S1) was sorted first using Fisher's two-sided p-value of association, a nd second using causality probability. From the top 20 ranked VNTRs, we manually selected on es where the target locus was previously linked to a phenotype. Top tissues are noted except w hen significance is seen in 4 or more tissues.

File Name: Supplementary Software 1.
Description: Software to genotype VNTRs from whole genome sequencing data.

File Name: Supplementary Software 2.
Description: Software to run eQTL analysis on gene expression levels.
