# Supplementary material for: Variable number tandem repeats mediate the expression of proximal genes
Source: Nat Commun. 2021 Apr 6;12:2075. doi: 10.1038/s41467-021-22206-z (PMC8024321; doi:10.1038/s41467-021-22206-z)
Supplement: Supplementary file 8 — Supplementary Software 1 [file 41467_2021_22206_MOESM8_ESM.zip › README_software_adVNTR-readthedocs-io-en-latest.pdf]

adVNTR Documentation

Release 1.1.1

Mehrdad Bakhtiari

May 25, 2020

Contents

1 Manuals **3**

2 Publication **11**

adVNTR is a tool for genotyping Variable Number Tandem Repeats (VNTR) from sequence data. It works with both NGS short reads (Illumina HiSeq) and SMRT reads (PacBio) and finds diploid repeating counts for VNTRs and identifies possible mutations in the VNTR sequences.

CHAPTER 1

Manuals

adVNTR is a tool for genotyping Variable Number Tandem Repeats (VNTR) from sequence data. It works with both NGS short reads (Illumina HiSeq) and SMRT reads (PacBio) and finds diploid repeating counts for VNTRs and identifies possible mutations in the VNTR sequences.

1.1 FAQ

- *How do I cite adVNTR?*
- *Can adVNTR work with repeating units that are shorter than 6bp?*
- *Can I run adVNTR to study expansion in other organisms?*
- *What sequencing platforms does adVNTR support?*

1.1.1 How do I cite adVNTR?

If you found adVNTR useful, we would appreciate it if you could cite our manuscript describing adVNTR:

Bakhtiari, M., Shleizer-Burko, S., Gymrek, M., Bansal, V. and Bafna, V., 2018. Targeted genotyping of variable number tandem repeats with adVNTR. Genome Research, 28(11), pp.1709-1719.

1.1.2 Can adVNTR work with repeating units that are shorter than 6bp?

Tandem repeats with period below 6bp are classified as Short Tandem Repeats (STRs). Although adVNTR can detect STRs expansions, we do not recommend to use it on STRs.

1.1.3 Can I run adVNTR to study expansion in other organisms?

You can run adVNTR for other organisms if you add custom VNTR to its database. However, it always returns diploid RU counts for the number of repeats and it is expected to get homozygous RU counts on haploid organisms.

1.1.4 What sequencing platforms does adVNTR support?

adVNTR is designed to analyze **Illumina** or **PacBio** sequencing data. We generally do not recommend to use it on sequencing data from other technologies as their error model is different.

1.2 Installation

In order to use adVNTR, it is recommended to (1) install adVNTR using conda packaging manager and (2) download the predefined models for human genome from *Data Requirements* section. However, you can install it from the source and/or use custom models.

1.2.1 Install adVNTR with conda

If you are using the conda packaging manager (e.g. miniconda or anaconda), you can install adVNTR from the bioconda channel:

```
conda config --add channels bioconda
conda install advntr
```

adVNTR could be invoked from command line with `advntr`

1.2.2 Data Requirements

In order to genotype VNTRs, you need to either train models for loci of interest or use pre-trained models (recommended): * To run adVNTR on trained VNTR models:

- Download vntr_data_recommended_loci.zip and extract it inside the project directory. This includes a set of pre-trained VNTR models in hg19 for Illumina (6719 loci) and Pacbio (8960 loci) sequencing data. You can use vntr_data_recommended_loci_hg38.zip for VNTRs in GRCh38.
- You can also download and use vntr_data_genic_loci.zip for 158522 VNTRs in hg19 that results in having much longer running time.

Alternatively, you can add model for custom VNTR. See *Add Custom VNTR* for more information about training models for custom VNTRs.

1.2.3 Execution:

Use following command to see the help for running the tool.

```
advntr --help
```

The program outputs the RU count genotypes of VNTRs. To specify a single VNTR by its ID use `--vntr_id <id>` option. The list of some known VNTRs and their ID is available at Disease-linked-VNTRs page in wiki.

See the demo execution here or *Quick Start* page to see an example data set with step-by-step genotyping commands.

1.2.4 Demo: input in BAM format

- `--alignment_file` specifies the alignment file containing mapped and unmapped reads:

```
advntr genotype --alignment_file aligned_illumina_reads.bam --working_directory ./log_
↪dir/
```

- With `--pacbio`, adVNTR assumes the alignment file contains PacBio sequencing data:

```
advntr genotype --alignment_file aligned_pacbio_reads.bam --working_directory ./log_
↪dir/ --pacbio
```

- Use `--frameshift` to find the possible frameshifts in VNTR:

```
advntr genotype --alignment_file aligned_illumina_reads.bam --working_directory ./log_
↪dir/ --frameshift
```

1.2.5 Install from source (Not recommended)

You can also install adVNTR from the source instead of using conda. First, you need to install the following packages:

Dependencies

1. **Following libraries are required to be installed on the system:**
    - `python2.7`
    - `python-pip`
    - `python-tk`
    - `libz-dev`
    - `samtools`
    - `muscle`

You can install these requirement in Ubuntu Linux by running `sudo apt-get install python2.7 python-pip python-tk libz-dev samtools muscle` To install these packages on Mac OS, you can use Homebrew.

2. **Following python2.7 packages are required:**
    - `biopython`
    - `pysam` version 0.9.1.4 or above
    - `cython`
    - `networkx` version 1.11
    - `scipy`
    - `joblib`
    - `scikit-learn`

You can install required python libraries by running `pip install -r requirements.txt`

To Install

To get the latest version and install it locally, run:

```
git clone https://github.com/mehrdadbakhtiari/adVNTR
cd adVNTR
make; make install
python setup.py install
```

adVNTR could be invoked from command line with `advntr`

Bakhtiari, M., Shleizer-Burko, S., Gymrek, M., Bansal, V. and Bafna, V., 2018. Targeted genotyping of variable number tandem repeats with adVNTR. Genome Research, 28(11), pp.1709-1719.

1.3 Quick Start

To help verify the installation and showing the workflow, we include a small data set and commands to genotype this simulated dataset. If you have already installed adVNTR, jump to *Genotype Predefined VNTR in Simulated Data*.

1.3.1 Install

The easiest way to get started is to *Install adVNTR with conda*. To install adVNTR, run these commands:

```
conda config --add channels bioconda
conda install advntr
```

1.3.2 Genotype Predefined VNTR in Simulated Data

To genotype a VNTR in the simulated dataset, one option is to use predefined models. Download vntr_data_recommended_loci.zip and extract it inside the project directory to use these models from human genome. Here, we genotype a VNTR with id 301645 that corresponds to a disease-linked VNTR. The list of some known VNTRs and their ID is available at Disease-linked-VNTRs page in wiki.

Then, download simulated sequencing data of a human sample. It only includes reads around a VNTR in CSTB gene which is known to be linked to progressive myoclonus epilepsies. Run this command to get 2/5 genotype for this VNTR.

```
advntr genotype --vntr_id 301645 --alignment_file CSTB_2_5_testdata.bam --working_
↪directory working_dir
```

1.3.3 Genotype Custom VNTR

You can train a new model for a VNTR that doesn't exist in predefined models. Instead of downloading vntr_data_recommended_loci.zip, you need the organism (here, human) reference genome to train a model for a specific VNTR. Download chromosome 21 of hg19 and extract it. It is recommended to have full reference genome of the organism to add the model, however, we use a single chromosome in quickstart since it is easier to download and runs faster. Run this command to add the VNTR in CSTB gene and train VNTR-specific scores:

```
advntr addmodel -r chr21.fa -p CGCGGGGCGGGG -s 45196324 -e 45196360 -c chr21
```

If you run the above command without using predefined models, this VNTR gets the first id. Run `genotype` command to get 2/5 genotype:

```
advntr genotype --vntr_id 1 --alignment_file CSTB_2_5_testdata.bam --working_
↪directory working_dir
```

1.4 Tutorial

1.4.1 Inputs

- NGS short reads (Illumina HiSeq)
- SMRT reads (PacBio)

1.4.2 Outputs

Currently there are two possible formats to get the genotyping output:

- **Text** Writes two lines in the output for each VNTR. The first contains the VNTR ID and the second line contains R1/R2 as the repeating unit counts. Below is an example output in text format for one VNTR:

  301645
  2/3

- **BED** BED format contains one line per locus and it is a tab-delimited output comprised of 9 columns: 1. The name of the chromosome, 2. Start position of the VNTR, 3. End position of the VNTR, 4. VNTR ID, 5. Name of the gene that contains the VNTR, 6. Repeating motif, 7. Number of repeats in reference genome, 8 and 9. Number of repeats in the sample. Below is an example output in BED format for one VNTR:

#CHROM	Start	End	VNTR_ID	Gene	Motif	RefCopy	R1	R2
chr21	45196324	45196360	301645	CSTB	CGCGGGGCGGGG	3	2	3

- **VCF** (Under construction)

1.4.3 Usage

adVNTR runs as follows:

```
usage: advntr <command> [options]
```

There are four commands:

genotype Determine repeat unit count and sequence variation within VNTRs.

viewmodel Show the structure and information about the VNTRs in the database.

addmodel Add a custom VNTR to the database.

delmodel Delete a VNTR from the database.

Each of these commands and their options is described below.

Genotype

Use `advntr genotype [options]` to genotype a VNTR using sequencing data. Alignment file and working directory are required.

Summary of options:

`--frameshift`: Use this option to identify frameshift instead of finding copy number of a VNTR.

`--pacbio`: Use this flag to genotype VNTRs using PacBio sequencing data.

`--update`: Use this option to iteratively update the model using real data before finding the genotype.

Input/output options:

-f, --alignment_file <file> alignment file in SAM/BAM/CRAM format

-r, --reference_filename <file> path to a FASTA-formatted reference file for CRAM files.

-f, --fasta <file> Fasta file containing raw reads

-p, --pacbio set this flag if input file contains PacBio reads instead of Illumina reads

-n, --nanopore set this flag if input file contains Nanopore MinION reads instead of Illumina

-o, --outfile <file> file to write results. adVNTR writes output to stdout if oufile is not specified

-of, --outfmt <format> output format. Allowed values are {text, bed} [text]

Algorithm options:

-fs, --frameshift set this flag to search for frameshifts in VNTR instead of copy number.

-e, --expansion set this flag to determine long expansion from PCR-free data

-c, --coverage <float> average sequencing coverage in PCR-free sequencing

--haploid set this flag if the organism is haploid

-naive, --naive use naive approach for PacBio reads

Other options:

-h, --help show this help message and exit

--working_directory <path> working directory for creating temporary files needed for computation

-m, --models <file> file containing VNTRs information [vntr_data/hg19_VNTRs.db]

-t, --threads <int> number of threads [4]

-u, --update set this flag to iteratively update the model

-vid, --vntr_id <text> comma-separated list of VNTR IDs

View VNTRs

Under construction . . .

Add Custom VNTR

Use `advntr addmodel [options]` to add a VNTR to the database. The structure of VNTR and its genomic coordinate are required.

Required arguments:

-r, --reference <text> Reference genome

-c, --chromosome <text> Chromosome (e.g. chr1)

-p, --pattern <text> First repeating pattern of VNTR in forward (5' to 3') direction

-s, --start <int> Start coordinate of VNTR in forward (5' to 3') direction

-e, --end <int> End coordinate of VNTR in forward (5' to 3') direction

Other options:

-g, --gene <text> Gene name

-a, --annotation <text> Annotation of VNTR region

-m/–models <file> VNTR models file [vntr_data/hg19_selected_VNTRs_Illumina.db] -h, –help show this help message and exit

You can use `--update` in genotyping step to iteratively update the model using real data.

Delete a VNTR

Use `advntr delmodel --vntr_id <ID>` to remove a VNTR from database.

Required arguments: -vid/–vntr_id <text> VNTR ID

Other options:

-m/–models <file> VNTR models file [vntr_data/hg19_selected_VNTRs_Illumina.db] -h, –help show this help message and exit

CHAPTER 2

Publication

Bakhtiari, M., Shleizer-Burko, S., Gymrek, M., Bansal, V. and Bafna, V., 2018. Targeted genotyping of variable number tandem repeats with adVNTR. Genome Research, 28(11), pp.1709-1719.
