# Supplementary material for: Variable number tandem repeats mediate the expression of proximal genes
Source: Nat Commun. 2021 Apr 6;12:2075. doi: 10.1038/s41467-021-22206-z (PMC8024321; doi:10.1038/s41467-021-22206-z)
Supplement: Supplementary file 8 — Supplementary Software 1 [file 41467_2021_22206_MOESM8_ESM.zip › adVNTR-master/docs/index.html]

xml version="1.0" encoding="utf-8" ?


# Welcome to adVNTR's documentation!

Contents:

System Message: ERROR/3 (/home/mehrdad/workspace/adVNTR/docs/index.rst, line 11)

Unknown directive type "toctree".

```
.. toctree::
   :maxdepth: 2
```

# Indices and tables

- :ref:`genindex`

  System Message: ERROR/3 (/home/mehrdad/workspace/adVNTR/docs/index.rst, line 19); *backlink*

  Unknown interpreted text role "ref".
- :ref:`modindex`

  System Message: ERROR/3 (/home/mehrdad/workspace/adVNTR/docs/index.rst, line 20); *backlink*

  Unknown interpreted text role "ref".
- :ref:`search`

  System Message: ERROR/3 (/home/mehrdad/workspace/adVNTR/docs/index.rst, line 21); *backlink*

  Unknown interpreted text role "ref".
