# Supplementary material for: Variable number tandem repeats mediate the expression of proximal genes
Source: Nat Commun. 2021 Apr 6;12:2075. doi: 10.1038/s41467-021-22206-z (PMC8024321; doi:10.1038/s41467-021-22206-z)
Supplement: Supplementary file 8 — Supplementary Software 1 [file 41467_2021_22206_MOESM8_ESM.zip › adVNTR-master/docs/_build/genindex.html]

Index — adVNTR 1.0.0 documentation


# Index

### Related Topics

- Documentation overview

### Quick search


Enter search terms or a module, class or function name.

©2018, Mehrdad Bakhtiari.
|
Powered by Sphinx 1.3.6
& Alabaster 0.7.7
