# Supplementary material for: Variable number tandem repeats mediate the expression of proximal genes
Source: Nat Commun. 2021 Apr 6;12:2075. doi: 10.1038/s41467-021-22206-z (PMC8024321; doi:10.1038/s41467-021-22206-z)
Supplement: Supplementary file 8 — Supplementary Software 1 [file 41467_2021_22206_MOESM8_ESM.zip › adVNTR-master/docs/_build/search.html]

Search — adVNTR 1.0.0 documentation


# Search

Please activate JavaScript to enable the search
functionality.

From here you can search these documents. Enter your search
words into the box below and click "search". Note that the search
function will automatically search for all of the words. Pages
containing fewer words won't appear in the result list.

### Related Topics

- Documentation overview

©2018, Mehrdad Bakhtiari.
|
Powered by Sphinx 1.3.6
& Alabaster 0.7.7
