# Supplementary figures and images for: Variable number tandem repeats mediate the expression of proximal genes
Source: Nat Commun. 2021 Apr 6;12:2075. doi: 10.1038/s41467-021-22206-z (PMC8024321; doi:10.1038/s41467-021-22206-z)

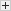

Supplement: Supplementary file 8 — Supplementary Software 1 [file 41467_2021_22206_MOESM8_ESM.zip › adVNTR-master/docs/_build/_static/plus.png]

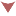

Supplement: Supplementary file 8 — Supplementary Software 1 [file 41467_2021_22206_MOESM8_ESM.zip › adVNTR-master/docs/_build/_static/down-pressed.png]

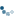

Supplement: Supplementary file 8 — Supplementary Software 1 [file 41467_2021_22206_MOESM8_ESM.zip › adVNTR-master/docs/_build/_static/ajax-loader.gif]

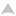

Supplement: Supplementary file 8 — Supplementary Software 1 [file 41467_2021_22206_MOESM8_ESM.zip › adVNTR-master/docs/_build/_static/up.png]

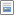

Supplement: Supplementary file 8 — Supplementary Software 1 [file 41467_2021_22206_MOESM8_ESM.zip › adVNTR-master/docs/_build/_static/file.png]

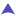

Supplement: Supplementary file 8 — Supplementary Software 1 [file 41467_2021_22206_MOESM8_ESM.zip › adVNTR-master/docs/_build/_static/up-pressed.png]

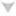

Supplement: Supplementary file 8 — Supplementary Software 1 [file 41467_2021_22206_MOESM8_ESM.zip › adVNTR-master/docs/_build/_static/down.png]

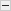

Supplement: Supplementary file 8 — Supplementary Software 1 [file 41467_2021_22206_MOESM8_ESM.zip › adVNTR-master/docs/_build/_static/minus.png]

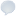

Supplement: Supplementary file 8 — Supplementary Software 1 [file 41467_2021_22206_MOESM8_ESM.zip › adVNTR-master/docs/_build/_static/comment.png]

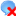

Supplement: Supplementary file 8 — Supplementary Software 1 [file 41467_2021_22206_MOESM8_ESM.zip › adVNTR-master/docs/_build/_static/comment-close.png]

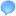

Supplement: Supplementary file 8 — Supplementary Software 1 [file 41467_2021_22206_MOESM8_ESM.zip › adVNTR-master/docs/_build/_static/comment-bright.png]
