# Supplementary material for: Variable number tandem repeats mediate the expression of proximal genes
Source: Nat Commun. 2021 Apr 6;12:2075. doi: 10.1038/s41467-021-22206-z (PMC8024321; doi:10.1038/s41467-021-22206-z)
Supplement: Supplementary file 9 — Supplementary Software 2 [file 41467_2021_22206_MOESM9_ESM.zip › README_software_VNTR-eQTL.pdf]

VNTR-eQTL

Scripts for analyzing VNTR-eQTLs in GTEx cohort (link to the paper) and genotyping performance comparisons

Inputs of the pipeline

1. VNTR genotypes of the entire GTEx cohort as outputted by adVNTR in the default format (one text file for each individual) using WGS data aligned to GRCh38 (available from dbGaP phs000424.v7.p2).
2. Phenotype file for GTEx cohort (ethnicity and sex are used). accession: pht002742.v7.p2 and exact version used in paper: phs000424.v7.pht002742.v7.p2.c1.GTEx_Subject_Phenotypes.GRU.txt
3. SNP genotypes file for GTEx cohort (phg001219.v1.GTEx_v8_WGS.genotype-calls-vcf.c1). This file is used to compare effect of VNTR variants and SNPs, and identifying population structure.
4. RNA-expression data for GTEx cohort in different tissues as a table (phe000020.v1.GTEx_RNAseq.expression-data-matrixfmt.c1).
5. SRA run table for GTEx expreiments downloaded from dbGaP. (used to identify corresponding tissue for each expreiment id)

Output of the pipeline

Linear regression results showing association of the length of each VNTR with the expression level of its nearest gene in each of the 46 tissues. The pipeline will create a directory called `regression_results` for the output. It contains 46 subdirectories (one for each tissue) and each of them have a tab separated file for each VNTR showing linear test for the VNTR in the corresponding tissue.

```
regression_results
└───Whole-Blood
│   │   100374.txt
│   │   100436.txt
│   │   ...
│
└───Lung
│   │   100374.txt
│   │   100436.txt
│   │   ...
│
└───etc
```

Each text file contains 6 tab separated values:

```
Genename  Chromosome  VNTR Coordinate Start   Effect Size (B)   P-value   Standard Error (Bse)
```

For example, `regression_results/Whole-Blood/423956.txt` (result of the association test for VNTR 423956 in Whole Blood) will have the following content:

```
POMC    chr2    25161573        0.21217799507799368     1.1501572764235368e-05  0.047746755235986996
```

Requirements

1. adVNTR: this can be installed with `conda config --add channels bioconda; conda install advntr`
2. eigensoft: this can be installed with `apt install eigensoft` on linux or `conda install -c bioconda/label/cf201901 eigensoft` using conda package manager on supported systems.
3. PEER: to correct for non genetic factors affecting gene expression level.
4. Python libraries: statsmodels, pandas, numpy
   These can be installed with `pip install pandas numpy statsmodels` using `pip` or with `conda install -c conda-forge statsmodels` using `conda`.
5. TRTools: for filtering target loci
6. CAVIAR: for fine-mapping the causal variant.

How to run

Preprocessing

1. From sra run table, we first extract the entries related to RNA-seq experiments:

```
zcat SraRunTable.txt.gz | grep "RNA-Seq" >  Sra_table_RNA-Seq_only
```

2. Using `Sra_table_RNA-Seq_only`, we then break up the rpkm expression matrix (phe000020.v1.GTEx_RNAseq.expression-data-matrixfmt.c1/GTEx_Data_20160115_v7_RNAseq_RNASeQCv1.1.8_gene_rpkm.gct) for different tissues:

```
python extract_expression_by_tissue.py Sra_table_RNA-Seq_only rpkm_file.gct Expression_by_Subtissue/
```

`Expression_by_Subtissue` will contain `Whole Blood.rpkm`, `Brain - Cortex.rpkm`, etc. This command usually takes an hour to complete.

3. To convert microarray genotypes to plink format and keep common variants to infer population structure, run following:

```
vcftools --gzvcf GTEx_Analysis_2017-06-05_v8_WholeGenomeSeq_866Indiv.vcf.gz --recode --maf 0.05 --remove-filtered-all --out filtere[illegible]
plink --vcf filtered_snps.vcf --biallelic-only --maf 0.05 --recode --out plink_866Ind_filtered_biallelic_snps_0.05maf
```

Since the vcf files are large, this step may take up to one day depending on the system.

Finding population structure

set `GTEXDIR` to parent directory of GTEx dataset in the `principal_component_identification.sh` scripts and run PCA and store results in `PCA_results` directory:

```
./principal_component_identification.sh PCA_results
```

Computing PEER factors

Run PEER factor identification scripts for each tissue (`Expression_by_Subtissue` directory was specified in preprocessing-part2).

```
python peer_factor_identification.py Expression_by_Subtissue PEER_results
```

This step may take 1-2 days to finish.

Running association test

This step will take less than a day to generate the `regression_results`

```
python run_regression.py VNTR_genotypes/ Expression_by_Subtissue/
```

`VNTR_genotypes` should contain 652 files (one per individual) as outputted by adVNTR for each sample.

Identifying significance threshold (5% FDR)

Run following script to read `regression_results` and identify tissue-specific significance thresholds.

```
compute_significance_cutoff.py
```
