# Supplementary material for: Variable number tandem repeats mediate the expression of proximal genes
Source: Nat Commun. 2021 Apr 6;12:2075. doi: 10.1038/s41467-021-22206-z (PMC8024321; doi:10.1038/s41467-021-22206-z)
Supplement: Supplementary file 10 — Reporting Summary [file 41467_2021_22206_MOESM10_ESM.pdf]

nature research

Corresponding author(s): Vineet Bafna

Last updated by author(s): Jan 8, 2021

# Reporting Summary

Nature Research wishes to improve the reproducibility of the work that we publish. This form provides structure for consistency and transparency in reporting. For further information on Nature Research policies, see our Editorial Policies and the Editorial Policy Checklist.

## Statistics

For all statistical analyses, confirm that the following items are present in the figure legend, table legend, main text, or Methods section.

| n/a | Confirmed | |
|---|---|---|
| ☐ | ☒ | The exact sample size ($n$) for each experimental group/condition, given as a discrete number and unit of measurement |
| ☐ | ☒ | A statement on whether measurements were taken from distinct samples or whether the same sample was measured repeatedly |
| ☐ | ☒ | The statistical test(s) used AND whether they are one- or two-sided<br>*Only common tests should be described solely by name; describe more complex techniques in the Methods section.* |
| ☐ | ☒ | A description of all covariates tested |
| ☐ | ☒ | A description of any assumptions or corrections, such as tests of normality and adjustment for multiple comparisons |
| ☒ | ☐ | A full description of the statistical parameters including central tendency (e.g. means) or other basic estimates (e.g. regression coefficient) AND variation (e.g. standard deviation) or associated estimates of uncertainty (e.g. confidence intervals) |
| ☐ | ☒ | For null hypothesis testing, the test statistic (e.g. $F$, $t$, $r$) with confidence intervals, effect sizes, degrees of freedom and $P$ value noted<br>*Give P values as exact values whenever suitable.* |
| ☐ | ☒ | For Bayesian analysis, information on the choice of priors and Markov chain Monte Carlo settings |
| ☒ | ☐ | For hierarchical and complex designs, identification of the appropriate level for tests and full reporting of outcomes |
| ☒ | ☐ | Estimates of effect sizes (e.g. Cohen's $d$, Pearson's $r$), indicating how they were calculated |

*Our web collection on statistics for biologists contains articles on many of the points above.*

## Software and code

Policy information about availability of computer code

| | |
|---|---|
| Data collection | We used fusera v1.0 software (https://github.com/mitre/fusera) to access GTEx data on AWS cloud. |
| Data analysis | We used our custom code adVNTR to genotype VNTRs which is an open-source software available on github (please refer to nr-software-policy_adVNTR form). We also used our own eQTL pipeline to analyze multiallelic tandem repeats which is also available on github (please refer to nr-software-policy_VNTR-eQTL form). |

For manuscripts utilizing custom algorithms or software that are central to the research but not yet described in published literature, software must be made available to editors and reviewers. We strongly encourage code deposition in a community repository (e.g. GitHub). See the Nature Research guidelines for submitting code & software for further information.

## Data

Policy information about availability of data

All manuscripts must include a data availability statement. This statement should provide the following information, where applicable:

- Accession codes, unique identifiers, or web links for publicly available datasets
- A list of figures that have associated raw data
- A description of any restrictions on data availability

All data used in this paper was not independently generated, but public data obtained through data use agreements with the providers. It is available to other users contigent on their independent agreements. We added accession codes and hyperlinks in Data Availability section and also in the manuscript where we describe each data. GTEx study data downloaded from the dbGaP web site, under phs000424.v7.p2 [https://www.ncbi.nlm.nih.gov/projects/gap/cgi-bin/study.cgi?study_id=phs000424.v7.p2]. The 30X whole genome sequencing data of 1000 Genomes Project samples used in this research were generated at the New York Genome Center with funds provided by NHGRI Grant 3UM1HG008901-03S1. This sequencing data is available at ENA Study PRJEB31736 [https://www.ebi.ac.uk/ena/browser/view/PRJEB31736] and ENA study PRJEB36890 [https://www.ebi.ac.uk/ena/browser/view/PRJEB36890]. RNA-seq data corresponding

to 465 samples from 1000 Genomes Project were downloaded from Geuvadis project [https://www.ebi.ac.uk/arrayexpress/experiments/E-GEUV-1/].The refseq data is vailable at UCSC Table Browser https://genome.ucsc.edu/cgi-bin/hgTables.

our analysis is available from: https://github.com/mehrdadbakhtiari/VNTR-eQTL/

# Field-specific reporting

Please select the one below that is the best fit for your research. If you are not sure, read the appropriate sections before making your selection.

[x] Life sciences [ ] Behavioural & social sciences [ ] Ecological, evolutionary & environmental sciences

For a reference copy of the document with all sections, see nature.com/documents/nr-reporting-summary-flat.pdf

# Life sciences study design

All studies must disclose on these points even when the disclosure is negative.

| | |
|---|---|
| Sample size | We used 3 independent datasets which we described in the paper. Briefly we use 652 sample from GTEx for our main findings. We use 462 samples from Geuvadis project and 902 samples from Icelandic cohort for our independent validation. |
| Data exclusions | It is described in the paper that some individuals,locus were excluded in some tissues where there was a missing data or other discrepancies (e.g. violating HWE for specific loci). We also excluded 12 individuals for eQTL tests from GTEx cohort that were excluded in the recent GTEx release hence their SNP microarray genotypes were missing in GTEx data and we could not fix covariates effect (population structure) for them. Sample ID of these individuals are as follows: GTEX-11DXY, GTEX-12BJ1, GTEX-13NYS, GTEX-13O1R, GTEX-14A5I, GTEX-14ICL, GTEX-14PHW, GTEX-16Z82, GTEX-OHPK, GTEX-QLQW, GTEX-YFCO, GTEX-ZVTK |
| Replication | We successfully replicated the findings in blood tissue as described in the manuscript. We attempted replication in two additional cohorts (geuvadis and icelandic) which were both successful and mentioned in the manuscript. |
| Randomization | We used permutation tests to correct for multiple testing and compute a 5% FDR rate.<br>We regressed out the effect of covariates in the regression analysis as described in methods.<br>We did not do any sample allocations and used all the samples in the GTEx dataset available to us. We also used all the samples for the two additional cohorts for the replication. |
| Blinding | The identity of the individuals were not known to us. We received the datasets from public repositories in an anonymized format.<br>We couldn't use blinding as we are associating the genetic variations with gene expression level for anonymized samples. |

# Reporting for specific materials, systems and methods

We require information from authors about some types of materials, experimental systems and methods used in many studies. Here, indicate whether each material, system or method listed is relevant to your study. If you are not sure if a list item applies to your research, read the appropriate section before selecting a response.

## Materials & experimental systems

| n/a | Involved in the study | |
|---|---|---|
| [x] | [ ] | Antibodies |
| [x] | [ ] | Eukaryotic cell lines |
| [x] | [ ] | Palaeontology and archaeology |
| [x] | [ ] | Animals and other organisms |
| [ ] | [x] | Human research participants |
| [x] | [ ] | Clinical data |
| [x] | [ ] | Dual use research of concern |

## Methods

| n/a | Involved in the study | |
|---|---|---|
| [x] | [ ] | ChIP-seq |
| [x] | [ ] | Flow cytometry |
| [x] | [ ] | MRI-based neuroimaging |

## Human research participants

Policy information about studies involving human research participants

| | |
|---|---|
| Population characteristics | We used public data from GTEx where of 86.0% of the donors were of European origin; 11.5% were African American and the remaining were Asian and American Indian. Our analysis regressed out covariate contributions (population structure through Principal Componenets, experimental covariates through PEER, age, and sex) as described in the manuscript. |
| Recruitment | We used human data from public sources. |
| Ethics oversight | The data was provided to the public with the appropriate approvals. |

Note that full information on the approval of the study protocol must also be provided in the manuscript.
